# Severe malnutrition or famine exposure in childhood and cardiometabolic non-communicable disease later in life: a systematic review

**DOI:** 10.1136/bmjgh-2020-003161

**Published:** 2021-03-10

**Authors:** Kelsey Grey, Gerard Bryan Gonzales, Mubarek Abera, Natasha Lelijveld, Debbie Thompson, Melkamu Berhane, Alemseged Abdissa, Tsinuel Girma, Marko Kerac

**Affiliations:** 1 Population Health, London School of Hygiene & Tropical Medicine, London, UK; 2 Division of Human Nutrition and Health, Wageningen University, Wageningen, Netherlands; 3 Department of Psychiatry, Jimma University, Jimma, Ethiopia; 4 Emergency Nutrition Network, Kidlington, Oxfordshire, UK; 5 Caribbean Institute for Health Research, University of the West Indies, Kingston, Jamaica; 6 Department of Pediatrics and Child Health, Jimma University, Jimma, Ethiopia; 7 Armauer Hansen Research Institute, Addis Ababa, Ethiopia; 8 Population Health, London School of Hygiene and Tropical Medicine, London, UK

**Keywords:** kwashiorkor, marasmus, cardiovascular disease, diabetes, nutritional and metabolic disorders

## Abstract

**Introduction:**

Child malnutrition (undernutrition) and adult non-communicable diseases (NCDs) are major global public health problems. While convincing evidence links prenatal malnutrition with increased risk of NCDs, less is known about the long-term sequelae of malnutrition in childhood. We therefore examined evidence of associations between postnatal malnutrition, encompassing documented severe childhood malnutrition in low/middle-income countries (LMICs) or famine exposure, and later-life cardiometabolic NCDs.

**Methods:**

Our peer-reviewed search strategy focused on ‘severe childhood malnutrition’, ‘LMICs’, ‘famine’, and ‘cardiometabolic NCDs’ to identify studies in Medline, Embase, Global Health, and the Cumulative Index to Nursing and Allied Health Literature (CINAHL) databases. We synthesised results narratively and assessed study quality with the UK National Institute for Health and Care Excellence checklist.

**Results:**

We identified 57 studies of cardiometabolic NCD outcomes in survivors of documented severe childhood malnutrition in LMICs (n=14) and historical famines (n=43). Exposure to severe malnutrition or famine in childhood was consistently associated with increased risk of cardiovascular disease (7/8 studies), hypertension (8/11), impaired glucose metabolism (15/24) and metabolic syndrome (6/6) in later life. Evidence for effects on lipid metabolism (6/11 null, 5/11 mixed findings), obesity (3/13 null, 5/13 increased risk, 5/13 decreased risk) and other outcomes was less consistent. Sex-specific differences were observed in some cohorts, with women consistently at higher risk of glucose metabolism disorders and metabolic syndrome.

**Conclusion:**

Severe malnutrition or famine during childhood is associated with increased risk of cardiometabolic NCDs, suggesting that developmental plasticity extends beyond prenatal life. Severe malnutrition in childhood thus has serious implications not only for acute morbidity and mortality but also for survivors’ long-term health. Heterogeneity across studies, confounding by prenatal malnutrition, and age effects in famine studies preclude firm conclusions on causality. Research to improve understanding of mechanisms linking postnatal malnutrition and NCDs is needed to inform policy and programming to improve the lifelong health of severe malnutrition survivors.

**Key questions**

**What is already known?**

- Many countries face a large ‘double burden’ of malnutrition: high prevalence of child undernutrition combined with a growing epidemic of overweight/obesity and associated cardiometabolic non-communicable diseases (NCDs).
- Convincing evidence for the Developmental Origins of Health and Disease hypothesis links prenatal malnutrition with increased long-term NCD risk, but less is known about the effects of severe malnutrition in childhood on NCD risk.

**What are the new findings?**

- Our review identified 57 studies examining NCD outcomes among survivors of historical famines (n=43) and severe childhood malnutrition (n=14).
- Severe malnutrition and famine exposure in childhood were consistently associated with increased risk of cardiovascular disease, impaired glucose metabolism, and metabolic syndrome (MetS). Some sex-specific effects were observed, with famine-exposed women at higher risk of glucose metabolism disorders and MetS.
- Heterogeneity across studies, uncontrolled confounding by prenatal malnutrition, and inadequate statistical adjustment for age effects in some famine studies were key limitations.

**Key questions**

**What do the new findings imply?**

- Preventing and treating severe malnutrition in children are not only important in their own right but also play a potentially important role in preventing NCDs. This is especially important in the context of impending global hunger related to the COVID-19 pandemic.
- Further research into mechanisms linking severe malnutrition in childhood with NCDs is needed to inform policy, programming, and patient management strategies that support long-term health in survivors of early-life malnutrition.

## Introduction

Severe malnutrition (undernutrition) in childhood and adult non-communicable diseases (NCDs) are two of the world’s most urgent public health problems.^1^ In all its forms, malnutrition accounts for some 45% of all mortality in children under 5 years.^2^ Severe malnutrition, particularly wasting, threatens the survival of an estimated 47 million children under 5 in low/middle-income countries (LMICs).^1^ In contrast, obesity-related NCDs are emerging as a leading cause of death in these settings, with nearly three quarters of all NCD deaths occurring in LMICs (28 million) including most premature deaths (82%).^3^ While convincing evidence for the Developmental Origins of Health and Disease (DOHaD) hypothesis links prenatal malnutrition with increased NCD risk later in life, less is known about the long-term sequelae of severe malnutrition during postnatal periods of developmental plasticity such as childhood and adolescence. However, it is biologically plausible that malnutrition during these crucial periods of postnatal growth and development may also have lasting effects on survivors’ health.^4^

At present, efforts to address severe childhood malnutrition are focused on community-based management with ready-to-use therapeutic foods along with inpatient treatment of complicated cases to prevent short-term mortality.^5^ As these efforts reduce case-fatality rates and global child mortality declines, considering the long-term health consequences of severe malnutrition and effects of therapeutic foods is increasingly important.^6 7^ Tackling NCDs is a priority under Sustainable Development Goal 3 (Good health and well-being), which aims to ‘reduce by one third premature mortality from NCDs through prevention and treatment’ by 2030.^8^ As LMICs face the financial and social penalties of the increasing NCD burden, it is imperative to prioritise NCD prevention. This area of research is especially topical as early evidence suggests that severe malnutrition in childhood may be linked with increased NCD risk for survivors.^9 10^

While a narrative review examining evidence of differences in cardiometabolic risk between marasmus and kwashiorkor survivors was conducted by Boyne *et al* in 2017, no systematic review examining evidence on NCD outcomes following severe malnutrition or famine exposure in childhood currently exists in the literature.^11^ As more children survive severe malnutrition globally, greater knowledge in this area is key to informing improved policy and programming around severe childhood malnutrition that reduce NCD risk. This review brings together evidence from studies of survivors of documented severe childhood malnutrition in LMICs or famines to present a synthesis of current knowledge on this topic.

## Methods

### Protocol and registration

This review follows the Preferred Reporting Items for Systematic Reviews and Meta-Analyses guidelines.^12^ The protocol was registered on PROSPERO (ID: CRD42019145683).

### Search strategy

A peer-reviewed search strategy focused around ‘severe childhood malnutrition, ‘LMICs’, ‘famine’, and ‘cardiometabolic NCDs’ was used to identify studies in Medline, Embase, Global Health, and CINAHL databases (search strategy in online supplemental file 1). Reference lists of studies identified through database searching were hand-searched for additional studies. All final searches were run on 31 July 2019.

10.1136/bmjgh-2020-003161.supp1 Supplementary data

### Eligibility criteria

Human studies published in English were assessed for eligibility against the following criteria:

*Population:* older children and adults who survived an episode of documented severe malnutrition in LMICs or famine exposure in childhood and adolescence (defined as 0–18 years of age).

*Exposure:* exposure definitions included two closely related groups: (1) documented severe malnutrition in childhood defined according to standard classifications based on low weight-for-height, low weight-for-age (WFA), low mid-upper arm circumference (MUAC), or nutritional oedema, or (2) famine conditions defined by severe food insecurity in the local area or country setting. Although severe stunting is a form of severe malnutrition, we excluded studies that considered stunted children alone as the association between stunting and increased NCD risk has been described elsewhere.^13^

*Comparators:* a comparison group unexposed to documented severe malnutrition or famine in childhood was preferred but not required.

*Outcomes:* a range of cardiometabolic NCD outcomes (eg, impaired glucose metabolism, dyslipidaemia, hypertension) was considered if they were based on an objective clinical outcome measured at least 1 year after exposure to severe malnutrition or famine. All study designs were eligible. Grey literature and unpublished studies were excluded.

### Screening and selection

Studies were screened for inclusion by a single author (KG) using a two-step process. First, potentially relevant studies were identified by screening titles and abstracts against the eligibility criteria. The full-text articles of identified studies were then reassessed to confirm their suitability for inclusion.

### Risk-of-bias assessment

A risk-of-bias assessment at study level was conducted using the appraisal checklist for quantitative studies reporting correlations and associations from the UK National Institute for Health and Care Excellence. This 16-item checklist facilitates assessment of a study’s internal and external validity (EV) based on key aspects of study design, including characteristics of study participants, definition of independent variables, outcomes assessed, and analytical methods.^14^ Each study is assigned an overall quality grade for internal validity and another for EV as follows: (··) all or most of the checklist criteria have been fulfilled, where they have not been fulfilled the conclusions are very unlikely to alter, (·) some of the checklist criteria have been fulfilled, where they have not been fulfilled, or not adequately described, the conclusions are unlikely to alter, or (**–**) few or no checklist criteria have been fulfilled and the conclusions are likely or very likely to alter.

### Data extraction, analysis, and reporting

Data were extracted using a standardised Microsoft Excel (2016) template that was piloted and adapted during the review process. The data extracted included: publication year, study design, study population, exposure definition, time since exposure, control group characteristics, outcomes, analytical methods, and key findings.

Due to the wide range of included NCD-related outcomes, meta-analysis was impossible. Therefore, a narrative synthesis was carried out for each outcome with a focus on any differential effects of exposure between subgroups where data allowed (eg, sex-specific or age-specific differences). Effect sizes for similar outcomes were compared across studies to identify areas of agreement or inconsistency in the results. Data from studies of famine survivors and documented severe childhood malnutrition were analysed separately to account for the different nature of the exposures.

### Patient and public involvement

Neither patients nor the public were involved in this research.

## Results

### Search results

**Figure 1:**
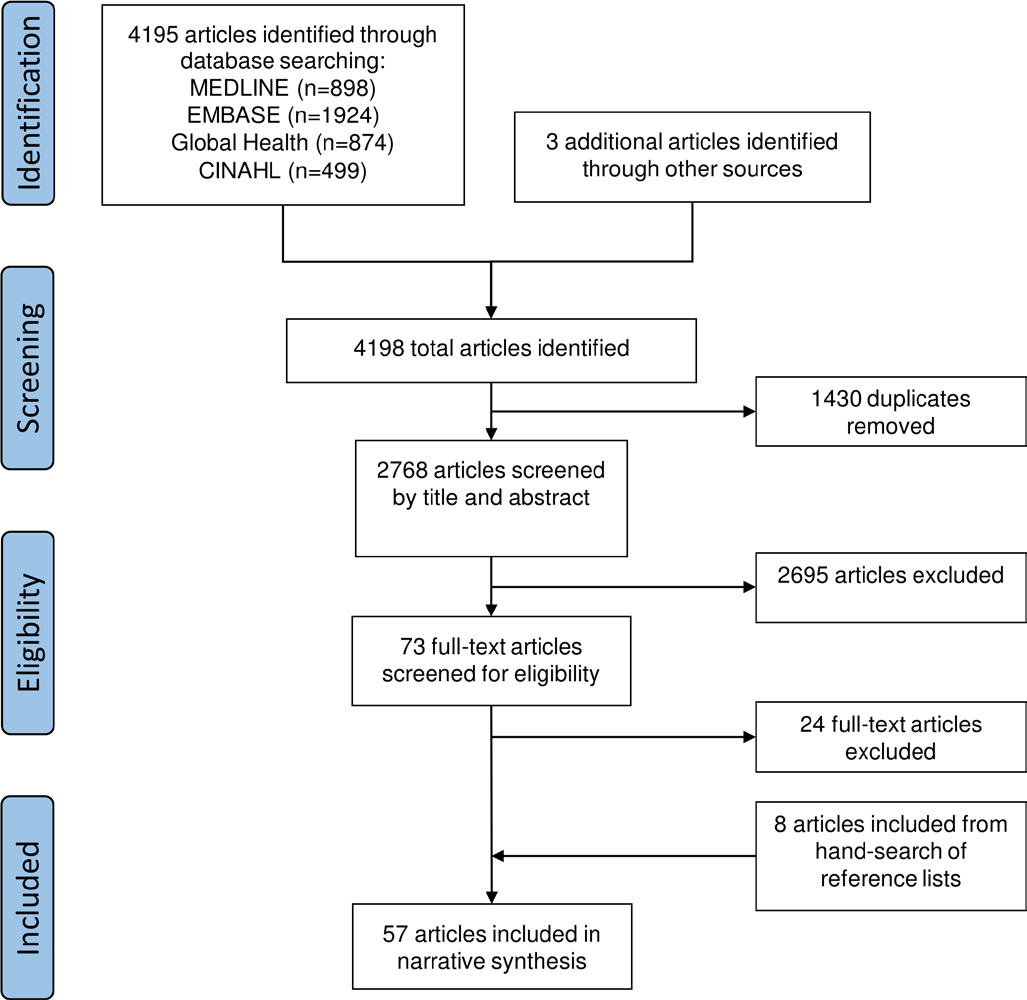
Preferred Reporting Items for Systematic Reviews and Meta-Analyses flow diagram of the study selection process.

The search yielded 2765 articles after removing duplicates. Three articles identified by senior authors were included for a total of 2768 articles for screening by title and abstract, which resulted in 73 articles for full-text appraisal. Another 24 articles were then excluded as they did not meet inclusion criteria. Eight eligible articles were identified from hand-searching reference lists for a total of 57 included articles (figure 1).

### Study characteristics

A total of 57 articles published between 1968 and 2019 were included, with 31 (54%) published between 2015 and 2019. Among these studies, 14 (25%) examined NCD outcomes among survivors of documented severe childhood malnutrition in LMICs and 43 (75%) studied famine survivors. Famine studies were predominantly retrospective cohorts (n=31; 72%), followed by prospective cohorts (n=7; 16%) and cross-sectional studies (n=5; 12%). Studies of documented severe malnutrition survivors were prospective cohorts (n=12; 86%) or case-control studies (n=2; 14%).

#### Study population and context

The studies represent findings from 15 countries; however, most were conducted among survivors of the Great Chinese Famine (n=30; 53%; table 1).

**Table 1:** Country and setting of included studies

Country	Setting	No. of studies
**Documented severe malnutrition studies**		
Jamaica	Tropical Metabolism Research Unit, Kingston	3
Senegal	Urban nutritional rehabilitation units, Thiès and Dakar	2
Malawi	Nutrition ward at central hospital, Blantyre	2
Uganda	Infantile Malnutrition Research Unit, Kampala	2
Mexico	Paediatric hospitals, Mexico City	2
Ethiopia	Urban health centres, Jimma and Gondar	1
Gambia	Medical Research Council field station, West Kiang	1
Kenya	Rural hospital, Kijabe	1
Total		**14**
**Famine studies**		
China	Great Chinese Famine (1959–1961)	30
The Netherlands	Dutch Hunger Winter (1944–1945)	4
Russia	Siege of Leningrad (1941–1944)	3
England	German occupation of Channel Islands (1944–1945)	2
Ukraine	Holodomor famine (1932–1933)	2
Bangladesh	Post-monsoon famine (1974–1975)	1
Nigeria	Biafran civil war (1967–1970)	1
Total		**43**

Participants in studies of documented severe childhood malnutrition were selected from clinic records of treatment for severe childhood malnutrition. However, in two case-control studies examining the exposure history of patients with diabetes, participants were recruited from outpatient clinics.^15 16^

Famine studies were conducted with participants exposed to famines between 1932 and 1970 (table 1). Participants in these studies were usually drawn from cross-sectional surveys or cohorts initiated for other studies. In some cases, participants were selected from physical examination records at health facilities or registries of patients with diabetes or siege survivors. In the Nigerian study, participants were recruited from central markets.^17^

In studies of documented severe childhood malnutrition, sample sizes ranged from 15 to 320 (median: 52; IQR: 34–100), whereas sample sizes varied between 62 and 105 374 for famine studies (median: 3548; IQR: 705–5920). For two of the famine studies, the number of cases and controls was not reported, and so the total number of participants was taken as the sample size.^18 19^ Gender balance among participants varied across studies from 30% to 83% female; however, three studies had male-only samples, six studies had only female participants and information on participant gender was unavailable for three studies.^20–22^

#### Definitions of exposure to severe childhood malnutrition and famine

Various criteria were used to define severe malnutrition exposure, with admission to nutritional rehabilitation units based on WHO, Wellcome and Gomez classifications being the most common. Three studies used clinical diagnosis of marasmus or kwashiorkor without precise definitions,^20 23 24^ and two used self-reports of severe childhood malnutrition.^15 16^ In the study by Moore *et al* (2001), childhood WFA z-scores for all participants were analysed against NCD outcomes regardless of whether they classified as malnourished.^25^

Famine exposure was most frequently defined according to participants’ birthdates and residency in famine-affected areas during childhood. However, five studies used individual self-reports of famine exposure.^26–30^ Excess mortality was often used to approximate levels of famine severity in different locations. Due to the different nature of the exposures, study characteristics have been presented separately for famine studies (table 2) and documented severe malnutrition studies (table 3) along with the results of the risk-of-bias assessment. Detailed tables containing effect sizes and *p*-values can be found in online supplemental file 2. An alternative version of table 2 categorising famine studies by study design is in online supplemental file 3.

10.1136/bmjgh-2020-003161.supp2 Supplementary data

10.1136/bmjgh-2020-003161.supp3 Supplementary data

**Table 2:** Summary of studies examining effects of early life famine exposure on non-communicable diseases (NCDs) outcomes

Study	Country and population	Exposure age group in years (sample size)	Outcome(s)	Key findings*	Risk-of-bias score (IV/EV)†
**Great Chinese Famine (1959–1961**)					
Chen *et al*^63^	China, adults>40 years	0–9 years (n=1799) 10–37 years (n=1064)	Visceral adipose dysfunction (VAD)	↑ VAD (women 0–9 years)	· / ·
Huang *et al*^35^	China, women~50 years	0–1.5 years (n=1035) 1.5–2.5 years (n=743)	Hypertension, body mass index (BMI)	↑ Hypertension (0–1.5 years) ↑ BMI (1.5–2.5 years)	· / ·
Li *et al*^44^	China, adults~45 years	0–2 years (n=1654) 3–5 years (n=1588) 6–8 years (n=1673)	Hyperglycaemia, type 2 diabetes (T2D)	↑ Fasting plasma glucose ↑ Hyperglycaemia (6–8 years)	· / ·
Li *et al*^58^	China, adults~45 years	0–2 years (n=1654) 3–5 years (n=1588) 6–8 years (n=1673)	Metabolic syndrome (MetS)	↑ MetS (0–2 years)	· / ·
Liu *et al*^19^	China, adults 35–74 years	0–9 years (n=n/r) 10–17 years (n=n/r)	Obesity	↑ Obesity	**–** / ·
Liu *et al*^40^	China, adults 45–53 years	0–3 years (n=455)	Hypertension	↔ Hypertension	**–** / **–**
Meng *et al*^51^	China, adults~45 years	1–3 years (n=31 363)	T2D, obesity	↔ T2D, obesity ↓ Abdominal obesity	· / ·
Shi *et al*^36^	China, adults~55 years	0–2 years (n=1149) 3–5 years (n=1217) 6–8 years (n=1250)	Cardiovascular disease (CVD)	↑CVD (with hypertension and famine exposure)	· / ·
Sun *et al*^48^	China, adults~55 years	1–3 years (n=1297) 4–6 years (n=1476) 7–10 years (n=1499)	Hyperglycaemia, T2D	↑ Hyperglycaemia (women) ↓ T2D (men 1–3 years, 7–10 years)	**–** / **–**
Wang *et al*^45^	China, adults~60 years	1–3 years (n=1932) 3–5 years (n=1712) 5–7 years (n=1953)	T2D, hyperglycaemia	↑ T2D, hyperglycaemic (women 3–5 years, 5–7 years)	· / **–**
Wang *et al*^46^	China, adults 52–93 years	0–9 years (n=1911) 10–37 years (n=1188)	T2D	↑ T2D (women, 0–9 years)	· / ··
Wang *et al*^97^	China, adults 52–93 years	0–9 years (n=1778) 10–37 years (n=1076)	Non-alcoholic fatty liver disease (NAFLD)	↑ NAFLD (women, 0–9 years)	·/··
Wang *et al*^47^	China, adults 52–77 years	0–9 years (n=1140) 10–33 years (n=706)	T2D	↑ T2D (0–9 years)	·/··
Wang *et al*^59^	China, adults 52–93 years	0–9 years (n=1776) 10–37 years (n=1053)	MetS	↑ MetS (women, 0–9 years)	·/··
Wang *et al*^98^	China, women 52–93 years	0–9 years (n=1679) 10–37 years (n=1003)	Chronic kidney disease (CKD)	↔ CKD	·/··
Wang *et al*^37^	China, adults~50 years	0–2 years (n=3126)	Hypertension, obesity	↑ Hypertension ↔ Obesity	·/··
Wang *et al*^37^	China, adults~50 years	1–3 years (n=4563)	Overweight, obesity	↑ Weight/BMI (women) ↑ Obesity (women)	**–** / **–**
Wang *et al*^38^	China, adults~50 years	0–1 years (n=338) 2–6 years (n=457)	Hypertension	↑ Hypertension (0–1 years)	· / ·
Wang *et al*^56^	China, adults~50 years	0–1 years (n=536) 2–6 years (n=597)	Dyslipidaemia	↑ Low-density lipoprotein cholesterol (women)	**–** /·
Wang *et al*^60^	China, adults~50 years	0–1 years (n=269) 2–6 years (n=717)	MetS	↑ MetS (0–1 years)	**–** /·
Woo *et al*^30^	China, adults>65 years	Age in childhood n/s (n=2222)	NCDs, blood pressure, BMI	↑ BMI, myocardial infarction ↔ T2D, hypertension	·/ **–**
Xin *et al*^57^	China, adults,~60 years	3–12 years (n=2132) 13–20 years (n=1140)	Dyslipidaemia	↑ Dyslipidaemia	**–** / **–**
Yao *et al*^55^	China, adults,~60 years	2–4 years (n=206)	Dyslipidaemia	↔ Dyslipidaemia	**–** / **–**
Yu *et al*^39^	China, adults~60 years	0–3 years (n=2115) 3–5 years (n=1941) 5–7 years (n=2248)	Hypertension	↑ Hypertension	· / **–**
Yu *et al*^61^	China, adults~60 years	0–3 years (n=1940) 3–5 years (n=1741) 5–7 years (n=2010)	MetS	↑ MetS (women)	· / **–**
Zhang *et al*^49^	China, adults~55 years	0–3 years (n=1582)	Hyperglycaemic, T2D	↑ Hyperglycaemia (women)	· / ·
Zheng *et al*^99^	China, adults~55 years	0–2 years (n=1344)	MetS	↑ MetS (women)	· / **–**
Zheng *et al*^62^	China, women~55 years	0–2 years (n=2403)	NAFLD	↑ NAFLD	· / **–**
Zheng *et al*^41^	China, adults~55 years	0–2 years (n=95)	Thyroid function	↓ Free thyroxine ↑ Thyroid stimulating hormone	**–** / **–**
Zhou *et al*^50^	China, adults 45–60 years	0–2 years (n=160) 3–5 years (n=173) 6–8 years (n=141)	NCDs	↑ T2D (0–2 years, 3–5 years) ↑ Hypercholesterolaemia (0–2 years)	**–** / **–**
**Dutch Hunger Winter (1944–1945)**					
Idris *et al*^29^	Netherlands, women~70 years	0–9 years (n=93) 10–18 years (n=54)	Coronary artery calcifications, valve calcification	↑ Coronary calcium score (10–18 years) ↔ Valve calcification	· / **–**
Portrait *et al*^31^	Netherlands, adults 60–76 years	0–1 years (n=81) 1–5 years (n=293) 6–10 years (n=244) 11–14 years (n=181)	Heart diseases, peripheral arterial diseases (PAD), T2D	↑ T2D, PAD (women, 11–14 years)	· / ·
van Abeelen *et al*^27^	Netherlands, women 49–70 years	0–9 years (n=n/r) 10–17 years (n=n/r)	T2D	↑ T2D (0–9 years)	· / ·
van Abeelen *et al*^28^	Netherlands, women 49–70 years	0–9 years (n=2196) 10–17 years (n=1773)	Coronary heart disease (CHD), stroke	↑ CHD (10–17 years) ↓ Stroke	· / ·
**Siege of Leningrad (1941–1944)**					
Koupil *et al*^33^	Russia, adults 40–70 years	1–5 years (n=81) 6–8 years (n=287) 9–15 years (n=739) 16–25 years (n=813)	CVD risk factors and mortality	↑ Hypertension (men 6–25 years) ↑ Ischaemic heart disease mortality (men 6–8 years) ↑ Cerebrovascular disease mortality (men 9–15 years)	· / ·
Rotar *et al*^34^	Russia, adults 64–81 years	0–1 years (n=50) 1–10 years (n=210)	Cardiovascular health, telomere length	↔ CVD, organ damage ↓ Telomere length	· / **–**
Sparen *et al*^32^	Russia, men 64 – 83 years	6–8 years, 9–15 years, 16–26 years (total n=1406)	CVD risk factors and mortality	↑ BP (9–15 years) ↑ Ischaemic heart disease mortality, stroke (9–15 years)	·· / ·
**German occupation of Channel Islands (1944–1945)**					
Head *et al*^22^	England, adults~70 years	8–22 years (n=225)	CVD	↑ CVD	**–** / **–**
Head *et al*^54^	England, adults~70 years	8–22 years (n=87)	Cholesterol levels	↔ Cholesterol levels	**–** / **–**
**Holodomor famine (1932–1933)**					
Khalangot *et al*^26^	Ukraine, adults>44 years	Age in childhood n/s (n=62)	Glucose tolerance	↓ T2D	· / ·
Vaiserman *et al*^18^	Ukraine, adults~70 years	0–3 years (n=n/r)	T2D	↔ T2D	· / ·
**Post-monsoon famine in Bangladesh (1974–1975)**					
Finer *et al*^21^	Bangladesh, adults~30 years	1–2 years (n=81)	Glucose tolerance, epigenetics	↔ Glucose tolerance ↕ Epigenetics	· / ·
**Biafran civil war (1967–1970)**					
Hult *et al*^17^	Nigeria, adults~40 years	0–3 years (n=246)	Hypertension, glucose tolerance, BMI	↑ Blood pressure ↔ Glucose tolerance, BMI	· / **–**

Acceptable IV and EV 
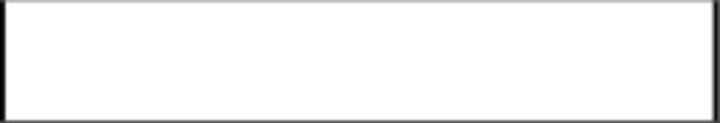
 Poor IV or EV 
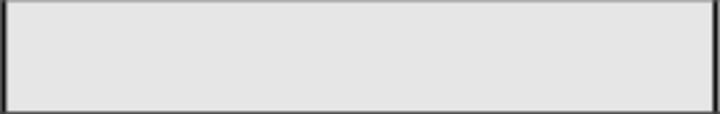
 Poor IV and EV 
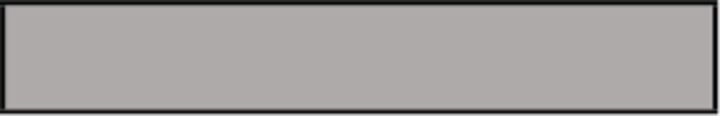
.

*Symbols for effect direction: ↑ increased; ↓ decreased; ↕ mixed (indicate statistically significant results were reported, defined as p<0.05); ↔ none (indicates no statistically significant result was reported). If no age group is indicated beside the finding, then all age groups were affected.

†The scoring system used in the risk-of-bias assessment is described in the Methods section.

**Table 3:** Summary of studies examining effects of documented severe malnutrition in childhood on non-communicable disease (NCD) outcomes

Study	Setting and population	Type/timing of severe malnutrition (SM) exposure	Outcomes	Key findings*	Risk-of-bias score (IV/EV)†
**Case-control studies**					
Chege^15^	*Cases*: patients with diabetes 61.8±10.9 years, Kenya (n=45) *Controls*: age and sex-matched non-diabetics from same area attending outpatient clinics (n=45)	Self-reported episode of SM in childhood Exposure age not specified	T2D risk factors	↑ Childhood SM among diabetics	**–** / **–**
Fekadu *et al*^16^	*Cases*: insulin-requiring diabetics 18–40 years, Ethiopia (n=107) *Controls*: age and sex-matched patients attending other hospital clinics (n=110)	Self-reported episode of childhood SM Exposure age not specified	Insulin-requiring diabetes risk factors	↑ Childhood SM in diabetics	·/ **–**
**Prospective cohort studies**					
Benefice *et al*^65^	*Ex-malnourished*: children 5.5±0.5 years, Senegal (n=52) *Chronic controls*: chronically undernourished children (n=54) *Well-nourished controls (WN**)*: age-matched, well-nourished children (n=33)	Marasmus Median age: 14 months	Motor fitness, anthropometry	↓ Handgrip in post-SM versus chronic controls ↓ Height/weight for age versus WN controls ↓ Distance throw, jump, agility/shuttle run versus WN controls ↔ Endurance run	·/ **–**
Boulé *et al*^52^	*Ex-malnourished*: young men 22.0±3.6 years, Mexico (n=26) *Controls*: young men 26.5±2.1 years with no history of SM (n=27)	Marasmus, kwashiorkor Age at admission: ≤1 years	Insulin sensitivity, abdominal obesity	↓ Insulin sensitivity w/ high abdominal fat versus fat-matched controls	· / **–**
Bourdon *et al*^77^	*Ex-malnourished*: children 9.6±1.6 years, Malawi (n=69) *Sibling controls*: closest in age to case child with no history of SM (n=44) *Community controls*: age and sex-matched (n=37)	Marasmus and kwashiorkor Median age at admission: 21.5 months	Cardiometabolic disease markers	↔ Metabolites	· / **–**
Cook^23^	*Ex-malnourished*: children 6.7–14.9 years, Uganda (n=31) *Controls*: children raised in similar environment as cases with no history of SM (n=21)	Kwashiorkor Mean age at admission: 1.9 years	Carbohydrate tolerance	↓ Glucose clearance ↑ Blood glucose 2 hours post oral glucose tolerance test (OGTT)	· / **–**
Francis-Emmanuel *et al*^53^	*Ex-malnourished*: adult marasmus survivors (MS) (n=42) and kwashiorkor survivors (KS) (n=38) 17–50 years, Jamaica *Community controls*: age, sex, BMI-matched (n=70) *Birthweight-matched controls*: age-matched (n=40)	Marasmus and kwashiorkor Age at admission: 6–18 months	Glucose metabolism	↔ Fasting plasma glucose ↑ Glucose intolerance (MS versus KS) ↓ Insulin sensitivity (MS versus KS) ↔ Insulin sensitivity (MS versus controls) ↓ Insulinogenic and oral disposition indices (MS vs all groups)	·· / **–**
Gonzalez-Barranco *et al*^42^	*Ex-malnourished*: young men 20.2±3.6 years, Mexico (n=52) *Controls*: young men with no history of SM (n=50)	Marasmus, kwashiorkor Mean age at admission: 4.5 months	Glucose metabolism, lipid profile, blood pressure (BP)	↑ Areas under the curves of glucose and insulin ↓ Insulin sensitivity, BP ↔ Fasting blood glucose, lipid profile	·· / **–**
Idohou-Dossou *et al*^20^	*Ex-malnourished*: children 6–8 years, Senegal (n=24) *Sibling controls (SC)*: closest in age to case child with no history of SM (n=24) *Well-nourished controls (WN)*: age-matched healthy children from wealthier area (n=19)	Marasmus Age at admission: 1–3 years	Biochemical nutritional indicators, growth factors, anthropometry	↓ Apolipoprotein A↔ versus WN controls, no difference between post-SM and SC ↓ Lean mass in post-SM and SC associated with low IGF-↔	· / **–**
Kajubi^24^	*Ex-malnourished*: adolescents 11–19 years, Uganda (n=15) *Controls*: adolescents with no history of SM (n=11)	Kwashiorkor Age at admission: 1.5–3 years	Pancreatic function	↔ Blood glucose post-OGTT ↓ Fasting plasma insulin	·/**–**
Lelijveld *et al*^9^	*Ex-malnourished*: children 9.6±1.6 years, Malawi (n=320) *Sibling controls (SC)*: closest in age to case child with no history of SM (n=217) *Community controls (CC)*: age and sex-matched with no history of SM (n=184)	Marasmus, kwashiorkor Median age at admission: 24 months	Blood markers of NCDs, physical capacity, anthropometry	↔ Glucose tolerance, glycosylated haemoglobin, blood lipids, salivary cortisol ↑ Diastolic BP in post-SM versus SC ↓ Handgrip strength versus CC/SC ↓ Lean mass versus CC but similar to SC	··/**–**
Moore *et al*^25^	Rural adults (mean age 35.8 years), Gambia (n=145)	Low weight-for-age z-score (WAZ) WAZ measured at 18 months	Cardiovascular disease (CVD) risk factors	↓ Fasting plasma insulin in lower WAZ quartiles ↔ Fasting blood glucose, blood glucose or insulin post-OGTT, cortisol, BP	··/**–**
Sheppard *et al*^84^	*Ex-malnourished*: adult survivors of kwashiorkor (KS) 29.82±9.03 years (n=21) or marasmus (MS) 25.02±5.69 years (n=23), Jamaica	Marasmus, kwashiorkor Mean age at admission: 11 months	Epigenetic profile in muscle tissue	↕ Differences in DNA methylation of 63 genes related to, body size/composition, glucose metabolism, musculoskeletal growth, cardiovascular pathways between MS and KS	·/**–**
Tennant *et al*^43^	*Ex-malnourished*: adult survivors of childhood kwashiorkor (n=62) 27.2±7.8 years and marasmus (n=54) 29.2±8.4 years, Jamaica *Community controls*: age and sex matched with no history of SM (n=45)	Marasmus, kwashiorkor Mean age at admission: 12 months	Cardiovascular structure/function	↓ Left ventricular outflow tract parameter, stroke volume, cardiac output, pulse wave velocity ↑ Diastolic BP ↔ Systolic BP ↑ Systemic vascular resistance ↑ Heart rate in MS versus KS ↔ Large vessel, cardiac remodelling	··/**–**

Acceptable IV and EV 
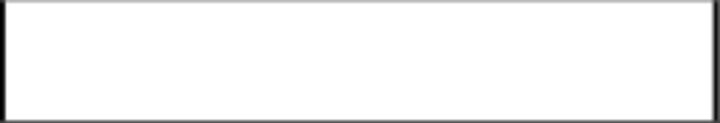
. Poor IV or EV 
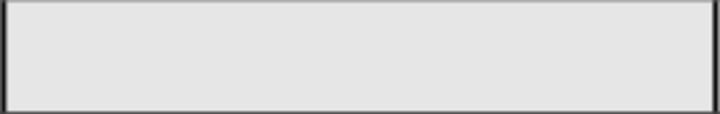
. Poor IV and EV 
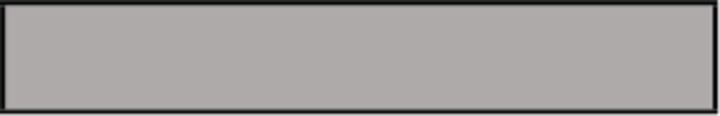
.

*Symbols for effect direction: ↑ increased; ↓ decreased; ↕ mixed (indicate statistically significant results were reported, defined as p<0.05); ↔ none (indicates no statistically significant result was reported). If no age group is indicated beside the finding, then all age groups were affected.

†The scoring system used in the risk-of-bias assessment is described in the Methods section.

T2D, type 2 diabetes.

#### Outcomes assessed

Included studies examined the impact of severe childhood malnutrition or famine exposure on one or more cardiometabolic NCD outcomes, including: glucose metabolism, cardiovascular disease (CVD), dyslipidaemia, non-alcoholic fatty liver disease (NAFLD), blood markers of cardiometabolic disease (eg, acute phase proteins, cortisol), physical capacity, metabolic syndrome, chronic kidney disease, epigenetic profile, telomere length, thyroid function, and anthropometry. Outcomes were assessed between 2 and 70 years post exposure in studies of documented severe malnutrition and 28–70 years post exposure in famine studies.

### Results by outcome

Because many of the studies presented in tables 2 and 3 reported findings on various outcomes, the results are summarised by outcome for clarity:

#### Cardiovascular outcomes

##### Famine studies

Famine exposure during childhood and adolescence was associated with increased risk of CVD (eg, myocardial infarction, coronary artery calcification) in 7/8 studies from the UK, China, Russia and the Netherlands,^22 27 29–33^ with only one study of Leningrad Siege survivors finding no difference in the prevalence of CVD.^34^ A sex-specific effect was noted in 2/3 studies; one study reported increased risk of peripheral arterial disease among women exposed to the Dutch Hunger Winter in adolescence, and another found higher mortality from cerebrovascular disease and ischaemic heart disease in men exposed to the Leningrad Siege at ages 9–15 years and 6–8 years, respectively.^31 33^

There was also consistent evidence of positive association between famine exposure and elevated blood pressure (BP) and hypertension in 8/11 studies from China, Nigeria and Russia.^17 32 33 35–39^ However, three Chinese studies found no difference in BP or hypertension risk between unexposed controls and those exposed to famine in infancy, early childhood, and late childhood, respectively.^30 40 41^ While the findings primarily indicate that famine exposure during infancy and childhood are associated with increased BP in adulthood, the two studies that included adolescents found positive associations between adolescent famine exposure (9–15 years) and increased BP.^32 33^

##### Documented severe malnutrition studies

Cardiovascular outcomes examined in studies of severe childhood malnutrition survivors largely focused on BP. These studies found mixed effects on BP outcomes, with one Jamaican study finding higher diastolic blood pressure (dBP) (d=4.3 mm Hg; p=0.007), but no differences in sBP, in survivors compared with controls at ~30 years, while a Mexican study found lower dBP (p=0.001) and sBP (p<0.0001) in survivors at ~20 years.^42 43^ Among Malawian survivors at ~9 years, dBP was higher than sibling controls (d=1.91 mm Hg, p=0.03).^9^ Meanwhile, a Gambian study showed no association between decreasing WAZ in the malnourished range in childhood and sBP or dBP at ~36 years in women.^25^

The single study that examined cardiovascular structure and function found reduced left ventricular outflow tract, stroke volume, cardiac output, and pulse wave velocity, together with increased systemic vascular resistance in survivors versus controls.^43^

#### Glucose metabolism outcomes

##### Famine studies

The evidence shows a positive association between childhood famine exposure and impaired glucose metabolism, with 2/15 studies finding increased risk of hyperglycaemia and 7/15 showing increased diabetes risk.^28 31 44–50^ The five studies that stratified analyses by sex found increased risk of glucose metabolism disorders in women only.^31 45 46 48 49^

Increased risk of hyperglycaemia in famine-exposed women was found in two Chinese studies. Both found similarly increased risk as a result of early childhood famine exposure (0–3 years; OR 1.48; 95% CI 1.15 to 1.9^48^; OR 1.55; 95% CI 1.10 to 2.19^49^), with one study finding further associations with exposure in mid-childhood (4–6 years; OR 1.38; 95% CI 1.06 to 1.79) and late childhood (7–10 years; OR 1.57; 95% CI 1.25 to 1.98).^48 49^ By contrast, one of these studies found a decreased risk of diabetes in men exposed to famine in early (OR 0.65; 95% CI 0.49 to 0.86) and late childhood (OR 0.74; 95% CI 0.56 to 0.98) compared with controls.^48^

Increased diabetes risk was found in seven studies from China and the Netherlands (5/7 and 2/7, respectively) after childhood famine exposure,^28 31 44–47 50^ with three studies reporting an effect in women only.^31 45 46^ Increased diabetes risk was mainly observed among participants exposed to famine in early and late childhood (0–10 years); however, one Dutch study reported increased risk in the female adolescent exposure group (11–14 years) only.^31^

Of the remaining six studies, five reported null findings and one reported a negative relationship between famine exposure and impaired glucose metabolism in adulthood. Studies from Bangladesh, Nigeria, China and Russia found no association between childhood famine exposure and impaired glucose metabolism when outcomes were assessed between ~30 and 80 years.^17 18 21 41 51^ Finally, one study of Ukrainian famine survivors reported reduced diabetes risk in childhood-exposed participants (OR 0.063; 95% CI 0.007 to 0.55) compared with unexposed controls.^26^

##### Documented severe malnutrition studies

The evidence indicates that severe childhood malnutrition is associated with impaired glucose metabolism in survivors, with 6/9 studies reporting a positive association with diabetes, reduced insulin sensitivity, or glucose intolerance.^15 16 23 42 52 53^ Notably, a Jamaican study that differentiated between survivors of marasmus and kwashiorkor reported greater fasting insulin, increased glucose intolerance, and reduced insulin sensitivity in adult marasmus survivors only.^53^ Conversely, a Ugandan study showed that glucose tolerance was impaired in kwashiorkor survivors compared with healthy controls.^23^ In a Mexican study, insulin sensitivity was reduced in survivors with high levels of abdominal fat even when matched to controls with similar levels of abdominal obesity; however, when matched for low amounts of abdominal fat, survivors and controls had similar insulin sensitivity.^52^ Severe childhood malnutrition was a risk factor for type 2 and insulin-requiring diabetes in case-control studies of patients with diabetes in Kenya and Ethiopia.^15 16^

The remaining 3/9 studies found no differences in glucose metabolism between severe malnutrition survivors and controls; however, these studies assessed outcomes in children, adolescents, and lean adults on a low-fat diet, respectively, which may have led to underestimation of the long-term effects of severe malnutrition.^9 24 25^

#### Lipid metabolism outcomes

##### Famine studies

Of eight studies that reported on lipid metabolism outcomes, four reported no difference in lipid profiles between famine-exposed participants and controls.^21 41 54 55^ Three Chinese studies found increased risk of dyslipidaemia after childhood famine exposure between 0 and 12 years, with one also reporting an effect in the adolescent group (13–20 years).^50 56 57^ A study of Leningrad Siege survivors found higher high-density lipoprotein (HDL) in exposed participants (p=0.008) but no difference in triglycerides compared with controls.^34^ Only one Chinese study stratified analyses by sex and it found an increased risk of dyslipidaemia in women only.^56^

##### Documented severe malnutrition studies

Three studies of severe malnutrition survivors examined lipid metabolism, with two finding no differences between the lipid profiles of controls and survivors at ~9 years (Malawi) and ~20 years (Mexico), respectively.^9 42^ One study found reduced apolipoprotein A1 in Senegalese marasmus survivors compared with well-nourished controls (p<0.05) but no difference compared with sibling controls.^20^

#### Metabolic syndrome outcomes

##### Famine studies

All five studies of metabolic syndrome (MetS) in Chinese famine survivors showed an increased risk in participants exposed between 0 and 9 years.^58–62^ Four studies stratified analyses by sex and found increased risk exclusively in women.^59–62^ Another Chinese study used a different method to assess MetS-related outcomes called the ‘visceral adiposity index’ (VAI), a sex-specific equation based on waist circumference, BMI, and triglyceride and HDL levels. They found a positive association between childhood famine exposure (0–9 years) and VAI in women only.^63^

#### Obesity-related outcomes

##### Famine studies

Evidence on obesity-related outcomes in famine survivors showed mixed effects. Four Chinese studies reported increased BMI, obesity or overweight among those exposed to famine between 0 and 9 years and followed up in late adulthood.^19 30 35 64^ Conversely, three studies found no association between famine exposure between 0 and 3 years and overweight, obesity, BMI or waist circumference.^17 37 41^

##### Documented severe malnutrition studies

In contrast with famine survivors, evidence on anthropometric outcomes in severe malnutrition survivors indicates that they remain thinner than unexposed controls through childhood to adulthood. Of six studies, five reported that survivors had lower BMI, WFA, MUAC or WAZ than controls when measured as older children or adults.^9 20 42 52 65^ However, two studies noted that WFA and WAZ were lower in severe malnutrition survivors compared with well-nourished controls but observed no difference when compared with chronically malnourished or sibling controls, respectively.^20 65^

Results for outcomes with ≤3 studies reporting results can be found in online supplemental file 4, including NAFLD, physical capacity, chronic kidney disease, thyroid function, metabolomics, and epigenetic and genetic outcomes.

10.1136/bmjgh-2020-003161.supp4 Supplementary data

## Discussion

### Summary of main results

We found evidence to support the hypothesis that exposure to severe malnutrition or famine during childhood increases long-term risk of cardiometabolic NCDs. The evidence was strongest for an association with CVD (myocardial infarction, coronary artery calcification, peripheral arterial disease, cerebrovascular disease, ischaemic heart disease, hypertension), impaired glucose metabolism (diabetes, hyperglycaemia) and MetS, while evidence for effects on lipid metabolism and obesity risk was less consistent. Where increased risk of NCDs in exposed groups was reported as an OR, effect sizes ranged from 1.11 to 5.50. Overall, these results suggest that childhood malnutrition may have a clinically, as well as statistically, significant effect on NCD risk in some survivors. Sex-specific differences were observed in some cohorts, with famine-exposed women at higher risk of glucose metabolism disorders and MetS than their male counterparts.

### Interpretation of findings

#### Windows of developmental plasticity

Current literature suggests that developmental plasticity extends beyond prenatal life and that severe malnutrition in childhood exerts independent effects on NCD risk. Due to heterogeneity in exposure age across studies, it is difficult to conclude whether morbidity risk is higher among children exposed at specific ages. However, it appears that the window of plasticity could remain open beyond the first 1000 days of life, which is the focus of much current child health policy and programming.

Our results build on a narrative review of differences in cardiometabolic risk between marasmus and kwashiorkor survivors from Boyne *et al* (2017) by systematically identifying new evidence for an effect of severe malnutrition and famine exposure in childhood on NCD risk.^11^ Building on the DOHaD hypothesis, these findings indicate that severe childhood malnutrition may not only have serious implications for short-term morbidity and mortality but also for survivors’ long-term health. This concept is described by Wells’ (2018) ‘capacity-load model of NCD risk’, which proposes that individuals develop physiological traits during early life that give them the capacity to maintain homeostasis in metabolism and cardiovascular function when challenged by a metabolic load.^66^ Therefore, if postnatal malnutrition impairs development of metabolic capacity, then survivors are more vulnerable to NCDs in later life, especially in an increasingly obesogenic environment.

#### Mechanistic links between severe malnutrition or famine in childhood and NCDS

There is little mechanistic evidence linking severe malnutrition or famine in childhood and long-term NCD risk, with most studies speculating on mechanisms or extrapolating from findings on prenatal malnutrition. Commonly proposed mechanisms include:

##### Growth acceleration

There is strong evidence that periods of rapid postnatal weight gain increase obesity and CVD risk later in life, with observational and intervention studies showing that accelerated early growth is associated with later body fatness as well as increased BP, cholesterolaemia and insulin resistance.^67–71^ Since rapid weight gain often follows episodes of severe childhood malnutrition, this may explain the increased CVD risk among those exposed to postnatal malnutrition followed by nutritional recovery.^72^ While the mechanisms linking rapid weight gain with NCD risk are not fully understood, the evidence is strongest for increased visceral adiposity as the key causal factor in CVD and diabetes.^73–75^

##### Body composition in later life

Endocrine changes caused by malnutrition may influence body composition in adult survivors and affect their NCD risk.^11^ Reduced growth factors (eg, IGF-1) and insulin, along with higher cortisol levels, may be conducive to stunting, reduced muscle mass, and a tendency towards obesity with high calorie intake.^76^ In studies of older children who experienced severe malnutrition in early life, survivors had less lean mass and more stunting compared with community controls, which was associated with low IGF-1.^9 20 77^ This phenotype may increase NCD risk as skeletal muscle is the major site of insulin-induced glucose uptake and therefore protects against insulin resistance and MetS.^78 79^

##### Impaired pancreatic function

Animal studies have shown that postnatal malnutrition negatively impacts pancreatic β-cell function.^80 81^ When malnutrition was induced in rats during lactation and the postweaning period using a low-protein diet, there were negative effects on insulin secretion leading to impaired glucose tolerance. On nutritional rehabilitation, the deleterious effects were reversed in the lactation-exposed group but not in the postweaning group, suggesting that postnatal malnutrition can permanently alter pancreatic function and lead to glucose metabolism disorders.^80^

##### Altered hypothalamic–pituitary–adrenocortical (HPA) axis

Exposure to stressors in utero and during childhood may alter the set-point of the HPA axis as an adaptation to cope with an anticipated high-stress environment in later life.^82^ However, these changes in neuroendocrine mediators of the stress response may predispose to metabolic disease when the adult environment is mismatched for these adaptations as excess glucocorticoids have been associated with hypertension and glucose intolerance.^83^

##### Epigenetic changes

While literature on epigenetic effects of early-life malnutrition on NCD risk largely focuses on the antenatal period, postnatal malnutrition may also cause epigenetic changes that contribute to future cardiometabolic disease risk.^21 84 85^ If epigenetic plasticity extends into postnatal life, then this may provide the mechanistic link between early-life malnutrition and later disease by ‘programming’ an adverse metabolic phenotype.^86 87^ However, epigenetic studies on the effects of childhood malnutrition are limited, and this theory requires further supporting evidence.

##### Sex-specific effects

Women exposed to famine in childhood appear to be at higher risk of glucose metabolism disorders and MetS than famine-exposed men. This finding is supported by a recent meta-analysis examining the effect of early-life famine exposure on risk of MetS in adulthood that included 39 studies (n=81 504). Compared with a non-exposed group, early-life famine exposure significantly increased the risk of MetS in women only.^88^ However, most studies were conducted in China where families may have preferentially allocated food and other resources to sons at the expense of daughters during the famine due to a culture of ‘son preference’.^89^ Malnutrition severity may thus explain increased NCD risk in women.^89^ While hypothetically this would select for the healthiest female survivors, it would also improve the average welfare of males, leading to better long-term health.^89^ Another explanation might be healthy survivor effect in boys as a recent review found that they are biologically more vulnerable to malnutrition and potentially only the healthiest survived.^90^

### Review limitations and strengths

We acknowledge several limitations. Due to our broad scope, there was extensive clinical and methodological heterogeneity between studies, rendering it difficult to directly compare study findings. As all studies were observational, associations cannot be interpreted as causal. This makes it difficult to disentangle the effects of fetal and postnatal malnutrition. Few studies controlled for effects of foetal malnutrition as the proxy measure of birth weight was rarely available; however, four studies showed independent effects of postnatal malnutrition on NCD risk after controlling for birth weight.^22 42 52 53^ Inadequately controlled age effects are another important confounder as most famine studies used birthdate to determine famine exposure. This was primarily an issue in Chinese studies as there were no truly unexposed areas during the Chinese famine, rendering it difficult to make comparisons with age-matched controls.^48^ Because there was no overlap in the birth years of exposed and non-exposed participants, age adjustment in regression models alone will have no impact on risk estimate calculations.^91^ Without an age-matched or age-balanced control group, age differences between groups may explain many of the effects on NCD outcomes attributed to famine exposure because ageing is a risk factor for NCDs and childhood-exposed groups were older than controls born post famine.^91 92^

Another limitation is the small sample size in some studies; thus, the children included may not represent the wider affected/at-risk population and chance observations are possible. We also note the risk of selection and information bias. Because severe childhood malnutrition is associated with high mortality, participants may represent the healthiest survivors and any effects observed in these populations may underestimate long-term health impacts of severe malnutrition.^9 93^ Misclassification of famine exposure status is an important source of information bias. Most studies assigned exposure status and severity based on birth date and place relative to famine years and regions of excess mortality. This may have resulted in misclassification of participants into incorrect exposure groups because individual exposure data were not available; participants may have been exposed to varying degrees of famine severity or even entirely protected for circumstantial reasons.

Finally, we recognise the currently limited data sources on this topic; although we identified an important number of individual papers, many were from the same famine event and some had high risk of bias. However, even when studies with higher risk of bias were discounted from our analysis, we found that most studies from the Great Chinese Famine, Dutch Hunger Winter and Siege of Leningrad (the famine events for which there were >2 available studies) showed a positive association between famine exposure and NCD outcomes. Therefore, our conclusions regarding the potential relationship between famine exposure and NCDs remain valid and all studies were included in the results so as to present a complete overview of available literature. We hope that future research in different settings might add further weight to our findings.

Our review has several strengths. To our knowledge, this is the only systematic review on this topic. It examines a highly topical subject; COVID-19 threatens to trigger food crises and famines worldwide, putting great numbers of children at risk of severe malnutrition.^94–96^ The resulting potential increase in NCDs risks putting a major burden on already overstretched health systems in LMICs in coming decades. Finally, while all studies were observational, there was consistency in findings, strength of associations, biological plausibility, temporal progression between exposure and outcomes, and coherence between epidemiological and laboratory findings.^80 81^

### Implications of findings and future research

If postnatal malnutrition influences long-term NCD risk as our results suggest, this is of global public health significance given the growing NCD epidemic in LMICs where there remains a large burden of severe childhood malnutrition.^3^ Prevention of severe malnutrition should be prioritised as no child should have to suffer the short-term or long-term effects of malnutrition. While short-term mortality and morbidity are widely recognised outcomes of malnutrition, we hope that the case for prevention will be further strengthened once policy-makers and funders appreciate the long-term sequelae highlighted in our review.

With over 80% of premature NCD-related deaths occurring in LMICs, where health systems already struggle with this massive disease burden, the need for interventions to prevent NCDs among severe malnutrition survivors is urgent. Our review highlights the limited evidence relevant to LMIC contexts, where severe malnutrition remains a threat to public health. Many of the highest quality studies we identified were conducted in settings where famine events were short lived and therefore may underestimate the effects of severe malnutrition in some contemporary contexts where malnutrition is endemic. There is a need for more high-quality studies in a wider range of contemporary LMIC settings to explore the potential links between severe malnutrition and NCDs.

There is reasonable strength and consistency in the findings and biological plausibility to indicate an association between severe malnutrition or famine exposure and increased NCD risk. However, the precise mechanisms underlying this association remain largely unclear. For instance, how do long-term outcomes differ by duration, intensity, and age at which a child experiences malnutrition and what are the biological processes leading to long-term adverse effects. Research in this area will be essential to inform policy and programming around prevention and management strategies for severe childhood malnutrition that promote long-term health in survivors as well as strategies to mitigate NCD risk among famine survivors. Recent predictions of major hunger following the COVID-19 pandemic make our review particularly timely.^94 95^ Taking action to prevent and appropriately treat this hunger is not only vital to save child lives, but also matters for NCD prevalence in decades to come.

## Conclusion

Our review indicates that severe malnutrition or famine exposure in childhood is associated with increased NCD risk later in life. The evidence on CVD, impaired glucose metabolism and MetS consistently shows deleterious effects of postnatal malnutrition on these chronic disease outcomes. Evidence for effects on lipid metabolism and obesity risk is less consistent.

Given that many countries with large burdens of child malnutrition also face NCD epidemics, understanding associations between severe childhood malnutrition and chronic diseases has major implications for preventing long-term morbidity and mortality. Increased global hunger resulting from the COVID-19 pandemic makes this link more important than ever. Efforts must be made to prevent and appropriately treat child malnutrition: not only to avoid short-term mortality, but to avoid escalating an already overwhelming NCD burden in decades to come. Better evidence is required from contemporary LMIC contexts where severe malnutrition may be inflicting long-lasting damage on public health.
