# Supplementary material for: Severe malnutrition or famine exposure in childhood and cardiometabolic non-communicable disease later in life: a systematic review
Source: BMJ Glob Health. 2021 Mar 10;6(3):e003161. doi: 10.1136/bmjgh-2020-003161 (PMC7949429; doi:10.1136/bmjgh-2020-003161)
Supplement: Supplementary data [file bmjgh-2020-003161supp001.pdf]

Online supplementary file 1
Severe malnutrition or famine exposure in childhood and cardiometabolic non-communicable disease risk later in life: a systematic review (Grey K et al., 2020)

SEARCH STRATEGY:
Database: Ovid MEDLINE(R) and In-Process & Other Non-Indexed Citations and Daily <1946 to July 30, 2019> Search Strategy:
----
1. ((severe* or acute* or moderate*) adj3 (malnutrition or malnourished or undernutrition or undernourished or wasted or wasting)) (6321)
2. ((post natal or postnatal) adj3 undernutrition) (174)
3. kwashiorkor* (3005)
4. ((oedematous or edematous) adj2 malnutrition) (66)
5. (nutrition* oedema or nutrition* edema) (101)
6. marasm* (1152)
7. ((non oedematous or non edematous or nonoedematous or nonedematous) adj2 malnutrition) (10)
8. wasting (16802)
9. wasted (3224)
10. emaciat* (2371)
11. wasting syndrome* (3156)
12. ((protein or energy) adj2 malnutrition) (9757)
13. nutrition* depriv* (719)
14. underweight (9759)
15. starvation (32720)
16. famine* (2254)
17. (low adj1 (MUAC or mid upper arm circumference or weight for length or WFL or WLZ or weight for age or WFA or WAZ)) (265)
18. exp severe acute malnutrition/ (2753)
19. protein-energy malnutrition/ (7213)
20. wasting syndrome/ (1166)
21. starvation/ (9737)
22. (metabolic adj3 syndrome*) (53310)
23. dysmetabolic syndrome* (108)
24. insulin resistance syndrome* (1716)
25. glucose metaboli* disorder* (1083)
26. ((type 2 or type II or obes*) adj1 diabet*) (138876)
27. ((type 2 or type II or obes*) adj1 DM) (3464)
28. (diabetes adj2 (type 2 or type II)) (170899)
29. (adult adj2 diabet*) (2410)
30. ((maturity onset or late onset) adj1 diabet*) (2053)
31. glucose intoleran* (15708)
32. glucose toleran* (56836)
33. insulin resistan* (91557)
34. MODY (1193)
35. NIDDM (6899)
36. DMNID (9)
37. ((non insulin dependent or noninsulin dependent or non insulin?dependent or noninsulin?dependent) adj1 diabet*) (11252)
38. ((non insulin treated or noninsulin treated or non insulintreated or noninsulin?treated or non insulin or noninsulin) adj1 diabet*) (142)

39. hyperinsulin* (26197)
40. glucose homeostasis (12345)
41. glucose regulation (2383)
42. insulin sensitiv* (31712)
43. (fasting adj2 glucose) (38270)
44. hyperglyc?emi* (66898)
45. glycated h?emoglobin (38356)
46. HbA1c (29756)
47. (cardio metabolic disorder* or cardiometabolic disorder*) (550)
48. lipid metabolism (93948)
49. lipid* profile* (28445)
50. plasma lipid* (13195)
51. hypercholesterol?emia (43123)
52. dyslipid?emia* (34335)
53. hyperlipid?emia* (43311)
54. (non alcoholic fatty liver disease or nonalcoholic fatty liver disease) (18003)
55. NAFLD (11670)
56. fat* liver (38196)
57. (cardiovascular disease* or cardio vascular disease*) (241048)
58. coronary heart disease* (48413)
59. hypertension (462338)
60. blood pressure (427853)
61. atheroscleros* (119342)
62. ((cardiovascular or cardio vascular) adj2 (function* or structure* or risk*)) (100701)
63. (muscle adj1 (strength or function)) (40358)
64. physical capacity (1784)
65. grip strength (10168)
66. (hand adj2 strength) (14799)
67. exp diabetes mellitus, type 2/ (124121)
68. exp insulin resistance/ (78156)
69. exp hyperglycemia/ (34546)
70. exp dyslipidemias/ (76958)
71. hypertension/ (227123)
72. arteriosclerosis/ (56520)
73. cardiovascular diseases/ (139159)
74. (Africa or Asia or Caribbean or West Indies or South America or Latin America or Central America) (245079)
75. (Afghanistan or Albania or Algeria or Angola or Antigua or Barbuda or Argentina or Armenia or Armenian or Aruba or Azerbaijan or Bahrain or Bangladesh or Barbados or Benin or Byelarus or Byelorussian or Belarus or Belorussian or Belorussia or Belize or Bhutan or Bolivia or Bosnia or Herzegovina or Hercegovina or Botswana or Brasil or Brazil or Bulgaria or Burkina Faso or Burkina Fasso or Upper Volta or Burundi or Urundi or Cambodia or Khmer Republic or Kampuchea or Cameroon or Cameroons or Cameron or Camerons or Cape Verde or Central African Republic or Chad or Chile or China or Colombia or Comoros or Comoro Islands or Comores or Mayotte or Congo or Zaire or Costa Rica or Cote d'Ivoire or Ivory Coast or Croatia or Cuba or Cyprus or Czechoslovakia or Czech Republic or Slovakia or Slovak Republic or Djibouti or French Somaliland or Dominica or Dominican Republic or East Timor or East Timur or Timor Leste or Ecuador or Egypt or United Arab Republic or El Salvador or Eritrea or Estonia or Ethiopia or Fiji or Gabon or Gabonese Republic or Gambia or Gaza or Georgia Republic or Georgian Republic or Ghana or Gold Coast or Greece or Grenada or Guatemala or Guinea or Guam or Guiana or Guyana or Haiti

or Honduras or Hungary or India or Maldives or Indonesia or Iran or Iraq or Isle of Man or Jamaica or Jordan or Kazakhstan or Kazakh or Kenya or Kiribati or Korea or Kosovo or Kyrgyzstan or Kirghizia or Kyrgyz Republic or Kirghiz or Kirgizstan or Lao PDR or Laos or Latvia or Lebanon or Lesotho or Basutoland or Liberia or Libya or Lithuania or Macedonia or Madagascar or Malagasy Republic or Malaysia or Malaya or Malay or Sabah or Sarawak or Malawi or Nyasaland or Mali or Malta or Marshall Islands or Mauritania or Mauritius or Agalega Islands or Mexico or Micronesia or Middle East or Moldova or Moldovia or Moldovian or Mongolia or Montenegro or Morocco or Ifni or Mozambique or Myanmar or Myanma or Burma or Namibia or Nepal or Netherlands Antilles or New Caledonia or Nicaragua or Niger or Nigeria or Northern Mariana Islands or Oman or Muscat or Pakistan or Palau or Palestine or Panama or Paraguay or Peru or Philippines or Philipines or Phillipines or Phillippines or Poland or Portugal or Puerto Rico or Romania or Rumania or Roumania or Russia or Russian or Rwanda or Ruanda or Saint Kitts or St Kitts or Nevis or Saint Lucia or St Lucia or Saint Vincent or St Vincent or Grenadines or Samoa or Samoan Islands or Navigator Island or Navigator Islands or Sao Tome or Saudi Arabia or Senegal or Serbia or Montenegro or Seychelles or Sierra Leone or Slovenia or Sri Lanka or Ceylon or Solomon Islands or Somalia or South Africa or Sudan or Suriname or Surinam or Swaziland or Syria or Tajikistan or Tadzhikistan or Tadjikistan or Tadzhik or Tanzania or Thailand or Togo or Togolese Republic or Tonga or Trinidad or Tobago or Tunisia or Turkey or Turkmenistan or Turkmen or Uganda or Ukraine or Uruguay or USSR or Soviet Union or Union of Soviet Socialist Repu blics or Uzbekistan or Uzbek or Vanuatu or New Hebrides or Venezuela or Vietnam or Viet Nam or West Bank or Yemen or Yugoslavia or Zambia or Zimbabwe or Rhodesia) (3499931)

76. ((developing or less* developed or under developed or underdeveloped or middle income or low* income or underserved or under served or deprived or poor*) adj (countr* or nation? or population? or world)) (90208)
77. ((developing or less* developed or under developed or underdeveloped or middle income or low* income) adj (economy or economies)) (485)
78. (low* adj (gdp or gnp or gross domestic or gross national)) (231)
79. (low adj3 middle adj3 countr*) (12882)
80. (lmic or lmics or third world or lami countr*) (6290)
81. transitional countr* (154)
82. famine* (2254)
83. Dutch Hunger Winter (46)
84. Siege of Leningrad (30)
85. developing countries/ (72581)
86. 1 or 2 or 3 or 4 or 5 or 6 or 7 or 8 or 9 or 10 or 11 or 12 or 13 or 14 or 15 or 16 or 17 or 18 or 19 or 20 or 21 (80392)
87. 22 or 23 or 24 or 25 or 26 or 27 or 28 or 29 or 30 or 31 or 32 or 33 or 34 or 35 or 36 or 37 or 38 or 39 or 40 or 41 or 42 or 43 or 44 or 45 or 46 or 47 or 48 or 49 or 50 or 51 or 52 or 53 or 54 or 55 or 56 or 57 or 59 or 60 or 61 or 62 or 63 or 64 or 65 or 66 or 67 or 68 or 69 or 70 or 71 or 72 or 73 (1546841)
88. 74 or 75 or 76 or 77 or 78 or 79 or 80 or 81 or 82 or 83 or 84 or 85 (3644081)
89. 86 and 87 and 88 (1361)
90. limit 89 to (english language and humans) (898)
