# Supplementary material for: Severe malnutrition or famine exposure in childhood and cardiometabolic non-communicable disease later in life: a systematic review
Source: BMJ Glob Health. 2021 Mar 10;6(3):e003161. doi: 10.1136/bmjgh-2020-003161 (PMC7949429; doi:10.1136/bmjgh-2020-003161)
Supplement: Supplementary data [file bmjgh-2020-003161supp002.pdf]

Online supplementary file 2

Severe malnutrition or famine exposure in childhood on cardiometabolic non-communicable disease risk later in life: a systematic review (Grey K et al., 2020)

Table 2. Detailed summary of studies examining effects of early life famine exposure on NCD outcomes

Lead author, year, study design	Population, setting, sample size (%female)	Timing of famine exposure	Years post-exposure	Outcome(s)	Key findings
Chen 2019[31]<br><br>Retrospective cohort	Study population: adults>40y, SPECT China Survey (n=3569, ~57% across groups)<br><br>Controls: conceived post-famine (n=1726)	Prenatal: n=706<br><br>Childhood: 0-9y, n=1799<br><br>Adolescent/adult: 10-37y, n=1064	~50y	Visceral adipose dysfunction (VAD)	-Positive association between childhood famine exposure and VAD in women (B=0.13;0.02-0.24; p<0.05) vs non-exposed women<br><br>-No association in men<br><br>PreN: Higher VAD in prenatal exposed women vs non-exposed (p<0.05)<br><br>(BW: n/r)
Finer 2016[21]<br><br>Retrospective cohort	Study population: adults ~30y, cross-sectional survey in rural Bangladesh (n=121, n/a)<br><br>Epigenetics sub-sample (n=143)<br><br>Controls: conceived post-famine (n=70)<br><br>Older controls: exposed >16y (n=112), for comparison to a background population	Prenatal: n=40<br><br>Postnatal: 1-2y, n=81	~28y	Metabolic profile, DNA methylation of metastable epialleles (ME) sensitive to maternal nutrition	-No differences in BG post-OGTT, T2D, IFG, IGT, or fasting lipids between postnatal and unexposed group (all p>0.05)<br><br>PreN: hypomethylation at 7 ME vs postnatal and control groups (p=0.0003)<br><br>(BW: n/r)

Head 2008[22]<br><br>Retrospective cohort	Study population: birth cohort attended by community midwife on Guernsey, UK (1923-37) (n=225, 52%)<br><br>Controls: evacuated before German occupation (n=648)	Postnatal: 8-22y, n=225	~50y	CVD	-Higher CVD risk in postnatal group vs controls (HR2.52; 1.54-4.13)<br><br>-Higher risk of CVD in urban parishes (more severe food deprivation) vs rural parishes (p=0.01)<br><br>(BW: no association between BW and CVD (HR/kg increase BW:1.12; 0.70–1.78))
Head 2009[32]<br><br>Retrospective cohort	Study population: birth cohort attended by community midwife on Guernsey, UK (1923-37) (n=87, 52%)<br><br>Controls: evacuated before German occupation (n=309)	Postnatal: 8-22y, n=87	~30y	Cholesterol levels	No association between exposure to occupation (d=0.04mmol/; -0.26-0.33) for exposed group) and cholesterol levels<br><br>(BW: no association between BW (d=0.08mmol/l per kg increase;0.17-0.34) and cholesterol levels)
Huang 2010[33]<br><br>Retrospective cohort	Study population: women born 1957-63 from folic acid trial from 1993-96 in three Chinese provinces (n=19,719, 100%)<br><br>n, rural=32,732 (more severe famine exposure)<br>n, urban=2,293<br><br>Controls: conceived post-famine(n=15,306)	Prenatal: 1960,1961,1962 (n=6195)<br><br>Postnatal: 1957, 1.5-2.5y, n=743<br><br>Postnatal: 1958,0-1.5y, n=1035	~30y	Hypertension, height, BMI	-Higher risk of HT in 1958 rural women 3.97x vs controls (log odds: 1.38; 0.17–2.59)<br><br>-Reduced height in 1958 rural women (d=1.66cm; 0.63-2.69) due to exposure<br><br>-Increased BMI in 1957 women (d=0.92 kg/m2; 0.32-1.51) due to exposure<br><br>PreN: lost height in 1959 rural women (1.33cm; 0.46-2.19), reduced BMI in 1960 (-0.32kg/m2; -0.58, -0.08) and 1961 group (-0.30 kg/m2; -0.58, -0.02). No association w/ HT.<br><br>(BW: n/r)
Hult 2010[17]	Study population: adults born in southeast Nigeria 1965-73 working in Enugu,	Fetal/infant: n=292	~40y	Hypertension, glucose tolerance (GT), overweight	-Higher risk of high BP (sBP>140mmHG) in overweight adults famine-exposed in childhood (OR=3.95; 1.88-9.04) vs controls

Prospective cohort	former Biafran capital (n=538, ~30% across groups)<br><br>Controls: conceived post-famine (n=486)	Childhood: 0-3y, n=246			-No differences in GT, BMI in childhood exposed group vs controls<br><br>PreN: Fetal-infant exposure associated w/ elevated sBP (p<0.001), dBP (p<0.001), BG (p<0.05), waist circumference (p=0.001), systolic HT (OR2.87; 1.90–4.34), IGT (OR1.65; 1.02–2.69) and overweight (OR1.41; 1.03–1.93) vs controls<br><br>(BW: n/r)
Idris 2013[29]<br><br>Retrospective cohort	Study population: post-menopausal women from Prospect-EPIC cohort (Utrecht, Netherlands 1993-97) (n=147, 100%)<br><br>Controls: conceived post-famine (n=139)	Preadolescence: 0-9y, n=93<br><br>Adolescence: 10-18y, n=54	~60y	Coronary artery calcifications	-Higher risk for high coronary calcium score after severe famine in adolescence vs controls (OR4.62; 1.16-18.43)<br><br>-No association between childhood famine exposure and valve or aortic calcification (OR1.66; 0.69-4.10)<br><br>(BW: n/r)
Khalangot 2017[26]<br><br>Cross-sectional	Study population: rural adults >44y from villages near Kyiv, Ukraine (2013-14) (n=62, ~75% across groups)<br><br>Controls: born<1947, confirmed no starvation in family (n=11)	Born before famine (<1947), confirmed starvation (age at exposure unclear, n=62)	~67-80y (two famines)	Glucose tolerance, anthropometry	-Higher risk of T2D in controls vs childhood exposure group (OR=0.063; 0.007-0.55)<br><br>-Negative association between adult height (OR0.86; 0.76-0.97), neck circumference (OR0.73; 0.54-0.97) and childhood exposure<br><br>(BW: n/r)
Koupil 2007[60]<br><br>Cross-sectional	Study population: men born 1916-35, women born 1910-40 living in St. Petersburg (formerly Leningrad) between 1975-82 (n=2011,	Early-childhood: 1-5y, n=81<br><br>Late-childhood: 6-8y, n=287	~40y	Cardiovascular risk factors and mortality	-Higher sBP in women exposed to peak of starvation in late childhood (d=8.8; 0.1–17.5mmHg) and men in puberty (2.9; 0.7–5.0mmHg) vs controls of same age

	~27% in late childhood, puberty, adolescence groups, 100% in early childhood and adulthood groups)<br><br>Controls: unexposed to famine (n=3319)	Puberty: 9-15y, n=739<br><br>Adolescence: 16-25y, n=813<br><br>Adulthood: 26-31y, n=91			-Excess of HT in men exposed to famine at 6–25y (OR1.20; 1.03-1.39)<br><br>-Higher mortality from ischaemic heart disease in men exposed in late-childhood (HR1.89; 1.18-3.01) and cerebrovascular disease w/ puberty exposure (HR1.27; 1.07-1.55)<br><br>(BW: n/r)
Li 2010[34]<br><br>Retrospective cohort	Study population: rural adults born 1954-64 from nationally representative CNNHS 2002 (n=5920, ~53% across groups)<br><br>Controls: conceived post-famine (n=1954)	Prenatal: n=1005<br><br>Early-childhood: 0-2y, n=1654<br><br>Mid-childhood: 3-5y, n=1588<br><br>Late-childhood: 6-8y, n=1673	~40y	Hyperglycemia, T2D	-Higher FPG in early (p=0.037), mid- (p=0.059) and late childhood (p=0.008) w/ severe famine exposure<br><br>-No consistent effects on hyperglycemia and T2D w/ childhood exposure<br><br>PreN: Higher FPG (mean diff=0.20mmol/l, p=0.007) and hyperglycemia (OR3.92; 1.64-9.39) w/ severe prenatal exposure. No differences in less severely exposed areas.<br><br>(BW: n/r)
Li 2011[35]<br><br>Retrospective cohort	SP: rural adults born 1954-64 from nationally representative CNNHS 2002 (n=5920, ~53% across groups)<br><br>Controls: conceived post-famine(n=1954)	Prenatal: n=1005<br><br>Early-childhood: 0-2y, n=1654<br><br>Mid-childhood: 3-5y, n=1588<br><br>Late-childhood: 6-8y, n=1673	~40y	MetS	-Higher risk of MetS w/ severe famine exposure in early childhood (OR2.85; 1.19-6.83, p=0.019) vs controls<br><br>-No differences in less severely exposed areas (all p>0.05)<br><br>PreN: Higher risk of MetS w/ severe prenatal famine exposure (OR3.12; 1.24-7.89, p=0.016) vs controls<br><br>(BW: n/r)

Liu 2017[19]<br><br>Retrospective cohort	Study population: adults 35-74y from two population-based surveys in Qingdao, China (2006, 2009) (n=8185*, 62%)<br><br>Controls: conceived post-famine (n= n/r)	Prenatal/infant: n=n/r<br><br>Childhood: 0-9y, n=n/r<br><br>Adolescence: 10-17y, n=n/r	~50y	Obesity	-Higher risk of obesity in childhood (OR1.42; 1.11-1.82, p<0.01) and adolescence (OR1.86; 1.25-2.77, p<0.01) exposed vs controls<br><br>-Higher risk of obesity at highest weight in childhood (OR1.24; 1.02-1.49) and adolescence (OR1.64; 1.40-1.93) exposed vs controls<br><br>PreN: Higher risk of obesity in fetal/infant exposed (OR1.59; 1.24-2.03, p<0.001) vs controls<br><br>(BW: n/r)
Liu 2017[36]<br><br>Retrospective cohort	Study population: adult residents 45-53y in Chongqing City (n=754, ~46% across groups)<br><br>Controls: conceived post-famine (n=470)	Fetal/infant: n=299<br><br>Childhood: 0-3y, n=455	~50y	Hypertension	No difference in HT risk after childhood exposure vs controls<br><br>PreN: higher HT risk in fetal/infant group (OR1.79; 1.13-2.84) w/ stronger effect in women (OR2.34; 1.01-5.42) than men (OR1.67; 0.95-2.92)<br><br>(BW: n/r)
Meng 2018[57]<br><br>Prospective cohort	SP: non-diabetic participants born around famine years from China Kadoorie Biobank (n=50, 242, ~60% across groups)<br><br>Controls: conceived post-famine (n=38,588)	Fetal: n=18,879<br><br>Early-childhood: 1-3y, n=31,363	~50y	T2D, obesity patterns	No association between early childhood exposure and risk of T2D (HR0.95; 0.67–1.36) vs controls<br><br>PreN: Increased risk of T2D in fetal-exposed (HR1.25; 1.07–1.45) vs age-balanced controls. Association between abdominal obesity and T2D in fetal-exposed group (p for interaction=0.025), stronger in women (p=0.013) than men (p=0.699).<br><br>(BW: n/r)

Portrait 2011[51]<br><br>Retrospective cohort	Study population: adults from Longitudinal Aging Study Amsterdam, nationally representative Dutch cohort (n=278, 47% in exposed)<br><br>Controls: from rural areas not exposed to famine (n=521, 57%)	Fetal/infant: 0-1y, n=81<br><br>Childhood: 1-5y, n=293<br><br>Pre-adolescence: 6-10y, n=244<br><br>Adolescence: 11-14y, n=181	~60y	Heart diseases, peripheral arterial diseases (PAD), T2D	Higher T2D risk in women (p=0.021) and PAD (p=0.018) exposed in adolescence vs controls. No association in men.<br><br>(BW: n/r)
Rotar 2017[37]<br><br>Retrospective cohort	Study population: siege survivors born 1930-43 registered with Petersburg Primorski District Society (n=278, 73%)<br><br>Controls: age, sex-matched, not in Leningrad during siege (n=51)	Fetal: n=45<br><br>Newborn/infant: 0-1y, n=50<br><br>Childhood: 1-10y, n=210	~60y	Cardiovascular health, telomere length (TL)	-No differences in prevalence of CVD or target organ damage between groups (all p>0.05)<br><br>-Shorter TL in survivors (p<0.0001), with clear association with the period of famine in early life. Newborn/infant group had longer TL (T/S ratio=0.63; 0.31-0.81) vs childhood (0.46; 0.23-0.62) and fetal group (0.44; 0.19-0.57) (p=0.023)<br><br>-Survivors had lower height (p=0.007), weight (p=0.008) and higher HDL (p=0.008)<br><br>(BW: n/r)
Shi 2018[39]<br><br>Retrospective cohort	Study population: adults born 1954-64 from China Health and Retirement Longitudinal Study (CHARLS) baseline survey (2011-12) (n=4378, ~53% across groups)<br><br>Controls: conceived post-famine (n=1394)	Fetal: n=762<br><br>Early-childhood: 0-2y, n=1149<br><br>Mid-childhood: 3-5y, n=1217<br><br>Late-childhood: 6-8y, n=1250	~50y	CVD	-Higher risk of CVD w/ HT in childhood-exposed groups: late (1.69; 1.06–2.72), mid- (2.35; 1.44–3.83), and early childhood (2.48; 1.49–4.11) vs unexposed<br><br>-Risk gradient between HT and CVD across groups mainly in women, in urban areas, w/ central obesity

					PreN: Higher risk of CVD w/ HT in fetal group (3.35; 1.54–7.27) vs controls<br><br>(BW: n/r)
Sparen 2014[58]<br><br>Prospective cohort	Study population: men born 1916-35 living in St Petersburg randomly selected for health exams (1975-7), mortality followed until 1999 (n=1406, 0%)<br><br>Controls: men of same age living in St Petersburg in 1975, no siege exposure (n=2499)	Early-childhood: 6-8y<br><br>Puberty: 9-15y<br><br>Young-adulthood: 16-26y<br><br>(n=1406 all groups)	~33-60y	Cardiovascular risk factors, mortality	-Higher dBP (p=0.02) and sBP (p=0.0003) in puberty-exposed group<br><br>-Higher mortality from ischaemic heart disease (RR1.39; 1.07-1.79), stroke (1.67; 1.15-2.43) including haemorrhagic stroke (1.71; 0.90-3.22) w/ puberty exposure. Effect on mortality partly mediated via BP but not any other measured biological, behavioural, or social factor.<br><br>(BW: n/r)
Sun 2018[59]<br><br>Retrospective cohort	Study population: adults born 1949-66 from CHARLS baseline survey (2011) (n=5661, ~54% across groups)<br><br>Controls: conceived post-famine(n=1601)	Fetal: n=1389<br><br>Early-childhood: 1-3y, n=1297<br><br>Mid-childhood: 4-6y, n=1476<br><br>Late-childhood: 7-10y, n=1499	~50y	Hyperglycemia (HG), T2D	-Higher HG in women exposed in early (OR1.48; 1.15–1.90), middle (1.38; 1.06–1.79) and late childhood (1.57; 1.25–1.98). Association stronger in women who lived in rural areas <16y (more severe famine)<br><br>-Lower T2D risk in men for early (0.65; 0.49–0.86) and late childhood (0.74; 0.56–0.98) exposure vs controls<br><br>PreN: higher HG risk in fetal-exposed women (1.34; 1.04–1.74)<br><br>(BW: n/r)
Vaiserman 2013[18]<br><br>Retrospective cohort	Study populations:<br>(1)T2D patients in 2000 born 1930-38 from four Ukraine regions exposed to	-Fetal: n= n/r<br><br>-Childhood: 0-3y, n=n/r	~70y	T2D	-No association between childhood famine exposure and T2D

	severe famine (n=28,358*, 71%)<br><br>(2)T2D patients in 2008 born 1920-64 in Ukraine regions with different famine histories (n=105, 374*, 67%)<br><br>Controls: birth cohorts conceived post-famine (n= n/a)				-Higher prevalence of T2D in females than males overall (OR 1.48; 1.46-1.50), more pronounced in famine exposed<br><br>PreN: increased T2D risk (OR1.5; p<0.001) in men and women conceived during peak famine vs pre- and post-famine cohorts<br><br>(BW: n/r)
van Abeelen 2012[27]<br><br>Retrospective cohort	Study population: women 49-70y from Prospect-EPIC cohort recruited 1993-97 exposed to Dutch famine between 0-21y (n=3572, 100%)<br><br>Controls: women reported 'hardly' any hunger, weight loss during famine (n=3,572)	Childhood: 0-9y, n=n/r<br><br>Adolescence: 10-17 y, n=n/r<br><br>Young-adulthood: >18 y, n=n/r<br><br>*n by degree of famine exposure:<br>-Moderate (n=2,975)<br>-Severe (n=1290)	~50y	T2D	-Higher risk of T2D w/ severe childhood famine (HR2.06; 1.37-3.10)<br><br>-Dose-dependent T2D risk relative to unexposed women: moderate exposure (HR1.36; 1.09-1.70) and severe exposure (HR1.64; 1.26–2.14)<br><br>(BW: n/r)
van Abeelen 2012[28]<br><br>Retrospective cohort	Study population: women 49-70y from Prospect-EPIC cohort recruited 1993-97 exposed to Dutch famine between 0-21y (n=4268, 100%)<br><br>Controls: women reported 'hardly' any hunger, weight loss during famine (n=3577)	Childhood: 0-9y, n=2196<br><br>Adolescent: 10-17y, n=1773<br><br>Young-adulthood: >18y, n= 299	~50y	Coronary heart disease, stroke	-Higher CHD risk after severe famine in adolescence (HR1.38;1.03-1.84)<br><br>-Lower stroke risk in famine-exposed women (HR0.77;0.59-.99)<br><br>(BW: n/r)

Wang, J 2016[40]<br><br>Retrospective cohort	Study population: retirees 56-63y from Dongfeng Motor Corporation cohort in China (n=6863, 95% fetal, 85% early, 79% mid, 73% late childhood)<br><br>Controls: conceived post-famine (n=938, 92%)	Fetal: n=1266<br><br>Early-childhood: 1-3y, n=1932<br><br>Mid-childhood: 3-5y, n=1712<br><br>Late-childhood: 5-7y, n=1953	~50y	T2D, hyperglycemia	-Higher T2D risk after mid- (OR1.55; 1.16-2.06) and late childhood (OR1.40; 1.05-1.87) exposure in women vs controls. No association in men. Similar associations for HG risk.<br><br>PreN: No association between fetal famine and dysglycemia risk<br><br>(BW: n/r)
Wang, N 2015[41]<br><br>Retrospective cohort	Study population: men and women from SPECT-China 2014 survey (n=3844, ~58% across groups)<br><br>Controls: conceived post-famine (40-51y, n=1808) and (<39y, n=1245)	Fetal: n=745<br><br>Childhood: 0-9y, n=1911<br><br>Adolescent/young-adult: 10-37y, n=1188	~55y	T2D	-Higher T2D risk in childhood-exposed women (OR2.81; 1.59–4.97) vs controls. No association in men.<br><br>-Living in areas with high economic status increased diabetes risk in adulthood (OR1.46; 1.20–1.78)<br><br>PreN: Higher T2D risk in fetal-exposed men (OR1.64; 1.04–2.59)<br><br>(BW: n/r)
Wang, N 2016[42]<br><br>Retrospective cohort	Study population: adults from SPECT-China survey (n=3566, ~57% across groups)<br><br>Controls: conceived post-famine (n=1740)	Fetal: n=712<br><br>Childhood: 1-10y, n=1778<br><br>Adolescent/young-adult: 11-38y, n=1076	~50y	NAFLD	-Childhood-exposed women at higher risk of moderate-severe NAFLD (OR1.82; 1.35-2.46) vs controls. No association in men.<br><br>-Association between increased alanine aminotransferase and famine in childhood-exposed women (p<0.05)<br><br>PreN: Fetal famine increased risk of moderate-severe NAFLD (OR1.77; 1.22- 2.57) and increased alanine aminotransferase (p<0.05)<br><br>(BW: n/r)

Wang, N 2017[43]<br><br>Retrospective cohort	SP: adults in Anhui and Shanghai provinces from SPECT-China (n=2335, ~59% across groups,39% in severe adolescent exposed)<br><br>Controls: conceived post-famine (n=1632)	Fetal: n=489<br><br>Childhood: 1-10y, n=1140<br><br>Adolescent/young-adult: 11-33y, n=706	~55y	T2D	Higher T2D risk after severe childhood exposure (OR1.44; 1.06-1.97)<br><br>PreN: Higher T2D risk after severe fetal exposure (OR1.90; 1.12-3.21)<br><br>(BW: n/r)
Wang, N 2017[44]<br><br>Retrospective cohort	Study population: men and women from SPECT-China 2014 survey (n=3530, ~57% across groups)<br><br>Controls: conceived post-famine (40-51y, n=1719) and (<39y, n=1196)	Fetal: n=701<br><br>Childhood: 0-9y, n=1776<br><br>Adolescent/young-adult: 10-37y, n=1053	~55y	MetS	Higher MetS risk in childhood-exposed women (OR1.80; 1.22-2.67) vs older controls. No association in men.<br><br>PreN: Higher MetS risk in fetal-exposed women (OR1.47; 1.05-2.07)<br><br>(BW: n/r)
Wang, N 2018[45]<br><br>Retrospective cohort	Study population: women >40y from SPECT-China 2014-15 (n=3329, 100%)<br><br>Controls: conceived post-famine (n=1795)	Fetal: n=647<br><br>Childhood: 1-10y, n=1679<br><br>Adolescent/young-adult: 11-38y, n=1003	~55y	Chronic kidney disease (CKD)	No association between childhood (OR1.23; 0.52-2.90) and adolescence (OR1.18; 0.39-3.59) exposure and CKD risk vs controls<br><br>PreN: Fetal famine exposure associated with lower eGFR (B=−1.35; −2.67,−0.04) and increased CKD risk (OR2.42; 1.05-5.58) vs controls<br><br>(BW: n/r)
Wang, P 2012[46]<br><br>Retrospective cohort	Study population: men and women 46–53y from health survey (2010) in Guangdong, China (n=7193, 51%)<br><br>Controls: conceived post-famine (n=4872)	Fetal: by trimester, n=1156<br><br>Infancy-only: 0-2y, n=3126<br><br>Fetal+infancy: n=2911	~50y	Hypertension, short stature, obesity	-Higher HT risk w/ exposure in infancy only (OR1.83; 1.61–2.08) and in fetal/infancy exposed group (OR1.31; 1.14–1.51) vs controls<br><br>-Exposure to famine during infancy increased the risk of short stature (p<0.01) but not obesity.

					<u>PreN:</u> Higher HT risk w/ first trimester exposure only (OR1.36; 1.03–1.79). No difference if exposed during 1st and 2nd trimester only or all three trimesters without subsequent infancy exposure.<br><br>(BW: n/r)
Wang, Y 2010[46]<br><br>Retrospective cohort	<u>Study population</u>: adults born 1956–64 who had physical evaluations at public health centre of Chongqing Medical University, China (2006-08) (n=8619, ~25% in exposed groups)<br><br><u>Controls:</u> conceived post-famine (n=8,404, 48%)	Fetal: n=4,056<br><br>Early childhood: 1-3y, n=4,563	~50y	Overweight, obesity	-Higher weight and BMI, lower height in female toddler group than controls (p<0.05)<br><br>-Higher risk of overweight in females in toddler group (OR1.48; 1.28–1.68) vs fetal group (OR1.26; 1.08–1.45)<br><br>-Higher risk of obesity in females in toddler group (OR1.46; 1.28–1.68) vs controls<br><br>-No impact of famine on adult body weight in males<br><br><u>PreN:</u> Higher weight/BMI, lower height in fetal group vs controls (p<0.05)<br><br>(BW: n/r)
Wang, Z 2016[47]<br><br>Retrospective cohort	<u>Study population</u>: men and women >45y from CHARLS baseline survey (2011-12) (n=1394, ~50% across groups)<br><br><u>Controls:</u> conceived post-famine (n=572)	Fetal: n=599<br><br>Infant: 0-1y, n=338<br><br>Pre-schoolers: 2-6y, n=457	~45y	Hypertension	-Higher sBP in infant-exposed and preschool-exposed cohorts (p<0.05). No difference in dBP (p>0.05).<br><br>-Increased HT risk after severe famine exposure in infant group only (OR2.11; 1.18-3.77) vs controls<br><br>-No consistent association in less severely affected areas or other exposed cohorts in severely affected areas

					(BW: n/r)
Wang, Z 2017[48]<br><br>Retrospective cohort	Study population: men and women >45y from CHARLS baseline survey (2011-12) (n=1935, ~50% across groups)<br><br>Controls: conceived post-famine (n=822)	Fetal: n=797<br><br>Infant: 0-1y, n=536<br><br>Pre-schoolers: 2-6y, n=597	~45y	Dyslipidemia	Increased LDL in women exposed to severe famine as infants (OR1.75; 1.17-2.62) or pre-schoolers (OR1.63; 1.10-2.42) vs controls. No association in males.<br><br>PreN: Higher LDL-c in women fetal-exposed to severe famine (OR1.80; 1.26–2.57)<br><br>(BW: n/r)
Wang, Z 2019[49]<br><br>Retrospective cohort	Study population: men and women born 1956-64 from CHARLS baseline survey (2011-12) (n=1415, ~52% across exposed groups)<br><br>Controls: conceived post-famine (n=733, 60%)	Fetal: n=429<br><br>Infant: 0-1y, n=269<br><br>Pre-schoolers: 2-6y, n=717	~50y	MetS	-Higher MetS risk in infant-exposed group (OR1.83; 1.24-2.70) vs controls<br><br>-Higher risk of FPG in infant-exposed group (OR2.00; 1.32-3.04). Associations between other components of MetS and famine exposure not significant but showed positive trends.<br><br>(BW: n/r)
Woo 2010[30]<br><br>Cross-sectional	Study population: adults ≥ 65 years who attended a health check at Chinese University of Hong Kong (n=2222, 49%)<br><br>Controls: reported no famine exposure in childhood (n=1510)	Childhood: age n/s, n=2222	~60y	NCDs, grip strength, walking speed, stride length, blood pressure, anthropometry	-Participants exposed to famine in childhood were shorter (OR0.92; 0.86-0.99), had higher BMI (OR1.11; 1.03-1.19) and appendicular lean mass/height^2 (OR1.17; 1.06-1.29) (ie. lower height but similar appendicular mass as controls)<br>-Higher risk of recurrent falls (OR1.67; 1.17-2.37), myocardial infarction (OR1.43; 1.09-1.86), arthritis (OR1.26; 1.05-1.51) and back pain (OR1.31; 1.12-1.54) in exposed vs controls<br>-No difference in grip strength between groups (OR1.06; 0.98-1.14)

					(BW: n/r)
Xin 2019[50]<br><br>Retrospective cohort	Study population: adults born 1941-66 from CHNN (2009) (n=3705, ~53% across groups)<br><br>Controls: conceived post-famine (n=1138)	Fetal/infant: fetal-2y, n=433<br><br>Childhood: 3-12y, n=2132<br><br>Adolescent: 13-20y, n=1140	~50y	Dyslipidemia	Higher risk of dyslipidemia in childhood (OR1.44; 1.23–1.69) and adolescence exposed (OR1.41; 1.17–1.71) vs controls<br><br>PreN: Higher risk of dyslipidemia after fetal exposure (OR1.34; 1.05–1.70) vs controls<br><br>(BW: n/r)
Yao 2019[51]<br><br>Retrospective cohort	Study population: adults born 1955-65 who completed health checkup at hospital in Hefei, China (2013) (n=333, 51%)<br><br>Controls: conceived post-famine (n= 271)	Fetal: n=127<br><br>Early-childhood: 2-4y, n=206	~50y	Dyslipidemia	-No association between childhood famine exposure and dyslipidemia (OR0.97; 0.67-1.41, p=0.89)<br><br>PreN: Fetal exposure to famine increased dyslipidemia risk in adult women vs controls (OR2.00; 1.03–3.86). No association in men.<br><br>(BW: n/r)
Yu 2017[52]<br><br>Retrospective cohort	Study population: retirees born 1952-64 from Dongfeng Motor Corporation cohort in Shiyan, China (2013 follow-up) (n=7698, 94% fetal, 84% early, 80% mid, 73% late childhood)<br><br>Controls: conceived post-famine (n=1044, 91%)	Fetal: n=1394<br><br>Early-childhood: 0-3y, n=2115<br><br>Mid-childhood: 3-5y, n=1941<br><br>Late-childhood: 5-7y, n= 2248	~50y	Hypertension	-Higher HT risk after famine exposure in early (OR1.44; 1.20–1.73), mid-(OR1.67; 1.38–2.02), and late childhood (OR2.11; 1.75–2.55) (P for trend<0.0001) vs controls<br><br>PreN: Higher HT risk after fetal famine exposure (OR1.24; 1.01–1.51) vs controls<br><br>(BW: n/r)
Yu 2018[53]<br><br>Retrospective cohort	Study population: retirees born 1952-64 from Dongfeng Motor Corporation cohort in Shiyan, China (2013 follow-up) (n=6959,	Fetal: n=1268<br><br>Early-childhood: 0-3y, n=1940	~50y	MetS	-Higher MetS risk in women exposed to famine in early (OR1.26; 1.02-1.56), mid- (OR1.43; 1.14-1.78) and late childhood (OR1.47; 1.18-

	95% fetal, 84% early, 80% mid, 73% late childhood)<br><br>Controls: conceived post-famine (n=956, 92%)	Mid-childhood: 3-5y, n=1741<br><br>Late-childhood: 5-7y, n=2010			1.84) vs controls. No association in men (P for famine-gender interaction = 0.0001).<br><br>(BW: n/r)
Zhang 2018[61]<br><br>Cross-sectional	Study population: adults born 1956-65 from chronic disease survey in northeastern China (2012) (n=3704, ~54% across groups)<br><br>Controls: conceived post-famine (n=1986)	Fetal: n=1442<br><br>Early-childhood: 0-3y, n=1582	~50y	Hyperglycemia, T2D	-Higher HG risk in women exposed in early childhood (OR1.55; 1.10–2.19) vs controls. No association in men.<br><br>PreN: Increased T2D risk in women in fetal-exposed group (OR1.67; 1.12–2.49) vs controls. No association in men.<br><br>(BW: n/r)
Zheng 2011[54]<br><br>Retrospective cohort	Study population: urban adults who underwent routine physical exams at Chongqing Medical University in China (2008) (n=2366, ~38% across groups)<br><br>Controls: conceived post-famine (n=2674)	Fetal: n=1022<br><br>Post-natal: 0-2y, n=1344	~45y	MetS	Higher MetS risk in postnatally exposed women (OR1.50;1.20-1.87, p=0.0003) vs controls. No association in men.<br><br>PreN: Higher MetS risk in prenatally exposed women (OR1.87; 1.15–3.04, p=0.012)<br><br>(BW: n/r)
Zheng 2017[55]<br><br>Retrospective cohort	Study population: urban women who underwent routine physical exams at Chongqing Medical University in China (2011-14) (n=4276, 100%)<br><br>Controls: conceived post-famine (n=4476)	Fetal: n=1873<br><br>Post-natal: 0-2y, n=2403	~50y	NAFLD	Higher risk of NAFLD in postnatally exposed women (OR1.26; 1.03-1.55) vs controls<br><br>PreN: Higher risk of NAFLD in prenatally exposed women (OR1.33; 1.04-1.70) and abnormal alanine aminotransferase (OR1.30; 1.05-1.61) vs controls<br><br>(BW: n/r)

Zheng 2019[56]<br><br>Retrospective cohort	Study population: urban adults who underwent routine physical exams at Chongqing Medical University in China (2017) (n=220, 61% fetal, 48% post-natal)<br><br>Controls: conceived post-famine (n=396, 57%)	Fetal: n=125<br><br>Post-natal: 0-2y, n=95	~60y	Thyroid function and nodules	-Lower FT4 and higher uTSH (p<0.05) in post-natal group vs controls<br><br>-No difference in numbers of thyroid nodules, TI-RADS score or maximal diameters of thyroid nodules<br><br>-No difference in heart rate or BMR between groups (p>0.05)<br><br>-No differences in BMI, waist circumference, BP, blood lipids, BG between groups (all p>0.05)<br><br>(BW: n/r)
Zhou 2019[62]<br><br>Cross-sectional	Study population: adults 45-60y recruited to medical centre for physical exams in Anhui, China (2011-12) (n=558, ~43% across groups)<br><br>Controls: conceived post-famine (n=381)	Fetal: n=84<br><br>Early-childhood: 0-2y, n=160<br><br>Mid-childhood: 3-5y, n=173<br><br>Late-childhood: 6-8y, n=141	~50y	Chronic diseases, dietary patterns	-Higher diabetes risk in early (PR3.13; 1.43-6.84) and mid-childhood (PR2.37; 1.05-5.36) groups vs controls<br>-Higher risk of hypercholesterolaemia in early childhood group (PR2.07; 1.01-4.25) vs controls<br>-Combination of early-life famine exposure and high-dichotomous high-fat/high-salt dietary pattern in adulthood increased risk for diabetes (PR4.95; 1.66-9.05) and hyper-cholesterolaemia (PR3.71; 1.73-7.60) compared to controls w/ low-dichotomous dietary pattern<br>(BW: n/r)

Abbreviations: **BG**: blood glucose; **BL**: blood lipids; **BP**: blood pressure; **BW**: birthweight; **CHD**: coronary heart disease; **CHARLS**: China Health and Retirement Longitudinal Study; **CKD**: chronic kidney disease; **CNNHS**: China National Nutrition and Health Survey; **dBP**: diastolic blood pressure; **eGFR**: glomerular filtration rate; **HDL**: high-density lipoprotein; **HG**: hyperglycemia; **IFG**: impaired fasting glucose; **IGT**: impaired glucose tolerance; **LDL**: low-density lipoprotein cholesterol; **MetS**: metabolic syndrome; **NAFLD**: non-alcoholic fatty liver disease; **n/r**: not reported; **n/s:** not specified; **OGTT**: oral glucose tolerance test; **PAD**: peripheral artery diseases; **sBP**: systolic blood pressure; **SPECT-China**: Survey on Prevalence in East China for Metabolic Diseases and Risk Factors in East China; **T2D**: type 2 diabetes

Table 3. Detailed summary of studies examining effects of documented severe childhood malnutrition on NCD outcomes

Lead author, year, study design	Population, setting, sample size (%female)	Type and timing of exposure to severe malnutrition	Age at follow-up	Outcome(s)	Key findings
Benefice 1999[63]<br><br>Prospective cohort	Study population: Children admitted to NRU for severe malnutrition (SM) 1988-1992 in Central Senegal (n=52, 48%)<br><br>Comparison: Chronically undernourished children from same area (n=54, 41%)<br><br>Controls: Age-matched, well-nourished (WN) (n=33, 52%)	Marasmus<br><br>Median age at admission:14m	5.5±0.5y	Motor fitness, anthropometry	-WN children taller, heavier for age than other groups (p<0.0001)<br><br>-WN group score better than chronic and SM groups in all outcomes except for endurance run (distance throw p<0.03, jump p<0.0001, agility/shuttle p<0.005, handgrip p<0.001)<br><br>-Reduced handgrip in SM vs chronic group, no other differences<br><br>-Stature is strongest predictor of motor performance, most differences between groups disappear after controlling for age and body size<br><br>(BW: n/r)
Boulé 2003[64]<br><br>Prospective cohort	Study population: young men admitted for SM treatment, Mexico City (n=26, 0%)<br><br>Controls: young men, no hx of SM (n=27, 0%)	Marasmus, kwashiorkor<br><br>Age at admission: ≤1y	SM: 22.0±3.6<br><br>Controls: 26.5±2.1	Insulin sensitivity (IS), abdominal obesity	-Negative association btw abdominal adipose tissue area and IS in both groups in post-SM and controls (r2=0.65 and 0.35, p<0.01)<br><br>-Similar insulin sensitivity when groups matched for low abdominal fat<br><br>-However, when matched for high amounts of abdominal fat, post-SM groups had lower insulin sensitivity (4.74 vs 6.85mgkg_1 min_1, P<0.05) than controls<br><br>(BW: not associated w/ IS or abdominal obesity)

Bourdon 2019[65]<br><br>Prospective cohort	Study population: Children treated for SM in 2006-2007 at central hospital in Malawi (n=69, 43%)<br><br>Sibling controls: closest in age, no hx of SM (n=44, 50%)<br><br>Community controls: age/sex matched (n=37, 41%)	Marasmus, kwashiorkor<br><br>Median age at admission: 21.5m	9.6±1.6y	Cardiometabolic disease markers (194 metabolites)	-No difference in metabolite profiles of survivors, siblings, and controls<br><br>-Current stunting associated w/ low IGF-1 and relationship modulated by SM (B=17.4, partial R^2=2.8%,p=0.025)<br><br>-Metabolites not associated w/ changes in WAZ or BMI-for-age since hospitalisation, severity, or type of SM<br><br>(BW: n/r)
Chege 2010[15]<br><br>Case-control	Study population: diabetic patients resident in rural Kenyan hospital's catchment area (n=45, 71%)<br><br>Controls: age/sex matched non-diabetics from same area attending outpatient clinics (n=45, 71%)	Self-reported childhood SM<br><br>Exposure age: n/s	61.8±10.9y	T2D risk factors	Childhood SM identified as T2D risk factor (RR2.08;1.20-3.61, p<0.009)<br><br>(BW: n/r)
Cook 1968[23]<br><br>Prospective cohort	Study population: inpatients cases of kwashiorkor at urban NRU in Uganda (n=31, 42%)<br><br>Controls: no hx of SM, raised in similar environment (n=21, 38%)	Kwashiorkor<br><br>Mean age at admission:1.9y	10.4y (6.7-14.9y)	Carbohydrate tolerance	-Rate of glucose clearance (%/min) for post-SM group (2.15±0.17) lower than controls (3.79±0.27), p<0.001<br><br>-Rise in BG 2hr post-OGTT (mg/100ml) higher in post-SM group (20.2±2.4) vs controls (10.5±3.7), p<0.05<br><br>(BW: n/r)

Fekadu 2010[16]<br><br>Case-control	Study population: insulin-requiring diabetics attending two urban health centres in Ethiopia (n=107, 27%)<br><br>Controls: age/sex-matched patients attending other hospital clinics (n=110, 32%)	Self-reported childhood SM<br><br>Exposure age: n/s	18-40y	Risk factors for insulin-requiring diabetes, anthropometry	-Diabetes associated w/ hx of childhood SM (OR=5.5; 1.0-29.0, p=0.047)<br><br>-Male diabetics shorter, lighter (p<0.001), with reduced sitting height (p<0.015), biacromial (p<0.003), and bitrochanteric (p< 0.008) diameters<br><br>(BW: n/r)
Francis-Emmanuel 2014[66]<br><br>Prospective cohort	Study population: adult Jamaican marasmus survivors (MS) (n=42, 42%) and kwashiorkor survivors (KS) (n=38, 47%) treated at urban NRU 1963-1992<br><br>Community controls (CC): age/sex/BMI-matched (n=70, 47%)<br><br>BW-matched controls (BWC): age-matched (n=40, 53%)	Marasmus, kwashiorkor<br><br>Age at admission:6-18m	17-50y	Glucose metabolism	-No difference in fasting glucose between groups (p>0.06)<br><br>-Glucose intolerance more common in MS (19%) than KS (3%), CC (11%), and BWC (10%) (OR10.9; 2.1–55, p=0.004, compared to KS)<br><br>-ISI lower in MS than KS (p<0.06) but similar between MS and controls. Insulinogenic index and oral disposition index lower in MS compared with all groups (p<0.01).<br><br>(BW: matched controls)
Gonzalez-Barranco 2003[67]<br><br>Prospective cohort	Study population: young men w/ hx of SM <1y recruited from four pediatric hospitals in Mexico City (n=52, 0%)<br><br>Controls: young men w/ no hx of SM (n=50, 0%)	Marasmus, kwashiorkor<br><br>Mean age at admission:4.5m	20.2±3.6y	Glucose metabolism, blood lipids, BP	-AUCG (p<0.012) and AUCI (p<0.002) higher in cases vs controls, ISI lower in cases (p<0.003) independent of BW, BMI, age<br><br>-No difference in FPL or FBG between groups (all p>0.1)<br><br>-Increasing BMI associated w/ higher FPI (p<0.006), AUCI (p<0.005), TGs (p<0.003), and lower HDL-C (p<0.006) and ISI (p<0.02) in cases but not controls

					-sBP (p<0.0001) and dBP (p=0.001) lower in cases than controls<br><br>(BW: not a predictor of any metabolic outcome)
Idohou-Dossou 2003[20]<br><br>Prospective cohort	Study population: children hospitalised for SM in poor suburb of Dakar, Senegal (n=24, n/a)<br><br>Sibling controls (SC): closest in age, no hx of malnutrition (n=24, n/a)<br><br>WN controls: age-matched healthy children from wealthier urban area (n=19, n/a)	Marasmus<br><br>Age at admission:1-3y	6-8y	Biochemical indicators of nutritional status, growth factors, anthropometry	-Apo A1 concentrations reduced in post-SM (p<0.05) and sibling controls (p<0.001) compared w/ WN children but no difference between post-SM and siblings. No difference in Apo B between groups.<br><br>-Mean anthropometrics of WN controls higher than post-SM and SC (p<0.001) but no difference between post-SM and SC<br><br>-FFM deficits in post-SM (p=0.014) and SC (p=0.019) associated w/ low IGF-1. HFA associated w/ IGF-1 in post-SM group only (p=0·026)<br><br>(BW: n/r)
Kajubi 1972[24]<br><br>Prospective cohort	Study population: adolescents admitted for SM as children to urban NRU in Kampala, Uganda (n=15, 33%)<br><br>Controls: adolescents w/ no hx of SM (n=11, 27%)	Kwashiorkor<br><br>Age at admission: 1.5-3y	11-19y	Pancreatic function	-Mean BG concentrations at all time points post-OGTT similar between cases and controls (all p>0.05)<br><br>-Fasting insulin higher in controls (p=0.05)<br><br>-Fasting concentrations of growth hormone lower in controls (p=0.05)<br><br>(BW: n/r)
Lelijveld 2016[9]<br><br>Prospective cohort	Study population: children admitted for SM to urban NRU in Blantyre, Malawi 2006-07 (n=320, 46%)	Marasmus, kwashiorkor<br><br>Median age at admission: 24m	9.6±1.6y	Blood markers of NCD risk, anthropometry, physical capacity, lung function	-No differences between survivors and controls for lung function, lipid profile, glucose tolerance, HbA1c, salivary cortisol, sitting height, head circumference (all p>0.05)<br><br>-dBP higher in survivors than SC (d=1.91mmHg, p=0·03)<br><br>-Weaker handgrip in post-SM group (adjusted diff vs CC –1.7kg;2.4 to –0.9,p<0.0001; adjusted diff vs SC

	Sibling controls: closest in age, no hx of SM (n=217, 51%)<br><br>Community controls: age, sex matched, no hx of SM (n=184, 48%)				1.01kg;0.3-1.7,p=0.005) and fewer minutes completed of exercise test (SC OR1.59;1.0-2.5,p=0·04; CC OR1.59;1.0-2.5,p=0·05)<br><br>-Post-SM had less lean mass than CC (adjusted diff vs CC –24.5, –43 to –5.5, p=0·01) but similar levels to SC after adjustment for age, (adjusted diff vs SC –11.5, –29 to –6, p=0.19)<br><br>(BW: n/r)
Moore 2001[25]<br><br>Prospective cohort	Study population: adults with known month of birth, born 1949-74 residing in three rural villages in The Gambia with detailed growth records available (n=145, 100%, WAZ analysis in females only)	Low WAZ<br><br>WAZ measured:18m	35.8y	CVD risk factors	-FPI marginally different between WAZ quartile groups (p=0.05) but no associations with any other risk factors for adult disease (fasting BG, p=0.85; 30min and 120min BG and insulin levels post-OGTT,all p>0.18; cortisol, p=0.2311; sBP, p=0.7579; dBP, p=0.81)<br><br>-Change in WAZ between early childhood and adulthood did not predict any measured risk factors<br><br>PreN data: no associations between season of birth (harvest vs hungry, proxy for fetal undernutrition) and any CVD risk factors<br><br>(BW: fetal undernutrition approximated by season of birth)
Sheppard 2017[68]<br><br>Prospective cohort	Study population: adult survivors of kwashiorkor (KS) (n=21, 56%) or marasmus (MS) (n=23 ,53%) in childhood treated at urban NRU in Kingston, Jamaica 1963-94	Marasmus, kwashiorkor<br><br>Mean age at admission:11m	KS:29.82±9.03y<br><br>MS:25.02±5.69y	Epigenetic profile in muscle	-Differences between KS and MS in methylation of CpG sites from 63 genes in skeletal muscle DNA related to immunity, body size and composition, glucose metabolism, musculoskeletal growth, neuronal function, and cardiovascular pathways<br><br>-Gene body is most likely region of variable methylation (OR:1.45, p=0.0036)

					-dmCpGs predominantly located on chromosome 6 (16% of total; OR2.4; p=0.0004); 10.5% each was found on chromosomes 7 and 17 (OR1.8; p=0·036 and OR1.9; p 0·021)<br><br>(BW: n/r)
Tennant 2014[69]<br><br>Prospective cohort	<u>Study population</u>: adult survivors of kwashiorkor (n=62, 34%) and marasmus (n=54, 46%) in childhood treated at urban NRU in Kingston, Jamaica 1963-99<br><br><u>Community controls:</u> age/sex matched, no hx of SAM (n=45, 60%)	Marasmus, kwashiorkor<br><br>Mean age at admission:12m	MS:29.2±8.4y<br><br>KS:27.2±7.8y	Cardiovascular structure and function	-Reduced left ventricular outflow tract parameter of 0.67 (0.16; p<0.0001), stroke volume 0.44 (0.17; p=0.009), cardiac output 0.5 (0.16; p=0.001) and pulse wave velocity 0.32 (0.15; p=0.03) in post-SM vs controls<br><br>-Higher dBP (d=4.3;1.2-7.3mmHg; p=0.007) in cases, sBP similar across groups<br><br>-No differences between KS and MS except heart rate (p=0.03)<br><br>-Systemic vascular resistance higher in post-SM, overall (5.5;2.8-8.4mmHg min/L; p<0.0001)<br><br>-No evidence of large vessel or cardiac remodeling or differences in other parameters<br><br>(BW: n/r)

<u>Abbreviations</u>: **AUCG**: areas under curves of glucose; **AUCI**: area under curves of insulin; **BL**: blood lipids; **BMI**: body mass index; **BW**: birthweight; **BWC**: birthweight-matched controls; **CC**: community controls; **CVD**: cardiovascular disease; **dBP**: diastolic blood pressure; **FBG**: fasting blood glucose; **FPI**: fasting plasma insulin; **FPL**: fasting plasma lipids; **GH**: growth hormone; **HbA1c**: glycated haemoglobin A1c; **HDL**: high-density lipoprotein cholesterol; **Hx**: history; **IGF-1**: insulin-like growth factor 1; **IGFBP-3**: insulin-like growth factor binding protein; **ISI**: insulin sensitivity index; **KS**: kwashiorkor survivor; **MS**: marasmus survivor; **n/r**: not reported; **NRU**: nutritional rehabilitation unit; **sBP**: systolic blood pressure; **SC**: sibling controls; **SES**: socio-economic status; **SM**: severe malnutrition; **SP**: study population; **TGs**: triglycerides; **Tx**: treatment; **WN**: well-nourished
