# Supplementary material for: Severe malnutrition or famine exposure in childhood and cardiometabolic non-communicable disease later in life: a systematic review
Source: BMJ Glob Health. 2021 Mar 10;6(3):e003161. doi: 10.1136/bmjgh-2020-003161 (PMC7949429; doi:10.1136/bmjgh-2020-003161)
Supplement: Supplementary data [file bmjgh-2020-003161supp003.pdf]

Table 1. Studies examining effects of early life famine exposure on NCD outcomes by study design

Study	Country and population	Exposure age group in years (sample size)	Outcome(s)	Key findings*	Risk of bias score (IV/EV)†
**Retrospective cohort studies**					
Chen *et al* 2019[31]	China, adults > 40y	0–9y (n=1799)<br>10–37y (n=1064)	Visceral adipose dysfunction (VAD)	↑ VAD (women 0–9y)	● / ●
Finer *et al* 2016[21]	Bangladesh, adults ~30y	1–2y (n=81)	Glucose tolerance, epigenetics	↔ Glucose tolerance<br>↕ Epigenetics	● / ●
Head *et al* 2008[22]	England, adults ~70y	8–22y (n=225)	Cardiovascular disease (CVD)	↑ CVD	– / –
Head *et al* 2009[32]	England, adults ~70y	8–22y (n=87)	Cholesterol levels	↔ Cholesterol levels	– / –
Huang *et al* 2010[33]	China, women ~50y	0–1.5y (n=1035)<br>1.5–2.5y (n=743)	Hypertension, body mass index (BMI)	↑ Hypertension (0–1.5y)<br>↑ BMI (1.5–2.5y)	● / ●
Idris *et al* 2013[29]	Netherlands, women ~70y	0–9y (n=93)<br>10–18y (n=54)	Coronary artery calcifications, valve calcification	↑ Coronary calcium score (10–18y)<br>↔ Valve calcification	● / –
Li, Y *et al* 2010[34]	China, adults ~45y	0–2y (n=1654)<br>3–5y (n=1588)<br>6–8y (n=1673)	Hyperglycemia, type 2 diabetes (T2D)	↑ Fasting plasma glucose<br>↑ Hyperglycemia (6–8y)	● / ●
Li, Y *et al* 2011[35]	China, adults ~45y	0–2y (n=1654)<br>3–5y (n=1588)<br>6–8y (n=1673)	Metabolic syndrome (MetS)	↑ MetS (0–2y)	● / ●
Liu, L *et al* 2017[19]	China, adults 35–74y	0–9y (n=n/r)<br>10–17y (n=n/r)	Obesity	↑ Obesity	– / ●
Liu, L *et al* 2017[36]	China, adults 45–53y	0–3y (n=455)	Hypertension	↔ Hypertension	– / –
Portrait *et al* 2011[37]	Netherlands, adults 60–76y	0–1y (n=81)<br>1–5y (n=293)<br>6–10y (n=244)<br>11–14y (n=181)	Heart diseases, peripheral arterial diseases (PAD), T2D	↑ T2D, PAD (women, 11–14y)	● / ●
Rotar *et al* 2017[38]	Russia, adults 64–81y	0–1y (n=50)<br>1–10y (n=210)	Cardiovascular health, telomere length	↔ CVD, organ damage<br>↓ Telomere length	● / –
Shi *et al* 2018[39]	China, adults ~55y	0–2y (n=1149)<br>3–5y (n=1217)<br>6–8y (n=1250)	CVD	↑CVD (with hypertension and famine exposure)	● / ●
Wang, J *et al* 2016[40]	China, adults ~60y	1–3y (n=1932)<br>3–5y (n=1712)<br>5–7y (n=1953)	T2D, hyperglycemia	↑ T2D, hyperglycemia (women 3–5y, 5–7y)	● / –
Wang, N *et al* 2015[41]	China, adults 52–93y	0–9y (n=1911)<br>10–37y (n=1188)	T2D	↑ T2D (women, 0–9y)	● / ●●
Wang, N *et al* 2016[42]	China, adults 52–93y	0–9y (n=1778)<br>10–37y (n=1076)	Non-alcoholic fatty liver disease (NAFLD)	↑ NAFLD (women, 0–9y)	● / ●●
Wang, N *et al* 2017[43]	China, adults 52–77y	0–9y (n=1140)<br>10–33y (n=706)	T2D	↑ T2D (0–9y)	● / ●●
Wang, N *et al* 2017[44]	China, adults 52–93y	0–9y (n=1776)<br>10–37y (n=1053)	MetS	↑ MetS (women, 0–9y)	● / ●●
Wang, N *et al* 2018[45]	China, women 52–93y	0–9y (n=1679)<br>10–37y (n=1003)	Chronic kidney disease (CKD)	↔ CKD	● / ●●
Wang, P *et al* 2012[46]	China, adults ~50y	0–2y (n=3126)	Hypertension, obesity	↑ Hypertension<br>↔ Obesity	● / ●●
Wang, Y *et al* 2010[46]	China, adults ~50y	1–3y (n=4,563)	Overweight, obesity	↑ Weight/BMI (women)<br>↑ Obesity (women)	– / –
Wang, Z *et al* 2016[47]	China, adults ~50y	0–1y (n=338)<br>2–6y (n=457)	Hypertension	↑ Hypertension (0–1y)	● / ●
Wang, Z *et al* 2017[48]	China, adults ~50y	0–1y (n=536)<br>2–6y (n=597)	Dyslipidemia	↑ Low-density lipoprotein cholesterol (women)	– / ●

Wang, Z *et al* 2019[49]	China, adults ~50y	0–1y (n=269)<br>2–6y (n=717)	MetS	↑ MetS (0–1y)	– / ●
Xin 2019[50] *et al*	China, adults, ~60y	3–12y (n=2132)<br>13–20y (n=1140)	Dyslipidemia	↑ Dyslipidemia	– / –
Yao *et al* 2019[51]	China, adults, ~60y	2–4y (n=206)	Dyslipidemia	↔ Dyslipidemia	– / –
Yu, C *et al* 2017[52]	China, adults ~60y	0–3y (n=2115)<br>3–5y (n=1941)<br>5–7y (n= 2248)	Hypertension	↑ Hypertension	● / –
Yu, C *et al* 2018[53]	China, adults ~60y	0–3y (n=1940)<br>3–5y (n=1741)<br>5–7y (n=2010)	MetS	↑ MetS (women)	● / –
Zheng, X *et al* 2011[54]	China, adults ~55y	0–2y (n=1344)	MetS	↑ MetS (women)	● / –
Zheng, X *et al* 2017[55]	China, women ~55y	0–2y (n=2403)	NAFLD	↑ NAFLD	● / –
Zheng, X *et al* 2019[56]	China, adults ~55y	0–2y (n=95)	Thyroid function	↓ Free thyroxine<br>↑ Thyroid stimulating hormone	– / –
**Prospective cohort studies**					
Hult *et al* 2010[17]	Nigeria, adults ~40y	0–3y (n=246)	Hypertension, glucose tolerance, BMI	↑ Blood pressure<br>↔ Glucose tolerance, BMI	● / –
Meng *et al* 2018[57]	China, adults ~45y	1–3y (n=31,363)	T2D, obesity	↔ T2D, obesity<br>↓ Abdominal obesity	● / ●
Sparen *et al* 2014[58]	Russia, men 64 – 83y	6–8y, 9–15y, 16–26y (total n=1406)	CVD risk factors and mortality	↑ Blood pressure (9–15y)<br>↑ Ischaemic heart disease mortality, stroke (9–15y)	●● / ●
Sun *et al* 2018[59]	China, adults ~55y	1–3y (n=1297)<br>4–6y (n=1476)<br>7–10y (n=1499)	Hyperglycemia, T2D	↑ Hyperglycemia (women)<br>↓ T2D (men 1–3y, 7–10y)	– / –
Vaiserman *et al* 2013[18]	Ukraine, adults ~70y	0–3y (n=n/r)	T2D	↔ T2D	● / ●
van Abeelen *et al* 2012[27]	Netherlands, women 49–70y	0–9y (n=n/r)<br>10–17y (n=n/r)	T2D	↑ T2D (0–9y)	● / ●
van Abeelen *et al* 2012[28]	Netherlands, women 49–70y	0–9y (n=2196)<br>10–17y (n=1773)	Coronary heart disease (CHD), stroke	↑ CHD (10–17y)<br>↓ Stroke	● / ●
**Cross-sectional studies**					
Khalangot *et al* 2017[26]	Ukraine, adults > 44y	Age in childhood n/s (n=62)	Glucose tolerance	↓ T2D	● / ●
Koupil *et al* 2007[60]	Russia, adults 40–70y	1–5y (n=81)<br>6–8y (n=287)<br>9–15y (n=739)<br>16–25y (n=813)	CVD risk factors and mortality	↑ Hypertension (men 6–25y)<br>↑ Ischaemic heart disease mortality (men 6–8y)<br>↑ Cerebrovascular disease mortality (men 9–15y)	● / ●
Woo *et al* 2010[30]	China, adults > 65y	Age in childhood n/s (n=2222)	NCDs, blood pressure, BMI	↑ BMI, myocardial infarction<br>↔ T2D, hypertension	● / –
Zhang *et al* 2018[61]	China, adults ~55y	0–3y (n=1582)	Hyperglycemia, T2D	↑ Hyperglycemia (women)	● / ●
Zhou *et al* 2019[62]	China, adults 45–60y	0–2y (n=160)<br>3–5y (n=173)<br>6–8y (n=141)	NCDs	↑ T2D (0–2y, 3–5y)<br>↑ Hypercholesterolaemia (0–2y)	– / –

* Symbols for effect direction: ↑increased; ↓decreased; ↕mixed (indicate statistically significant results were reported, defined as $p < 0.05$); ↔none (indicates no statistically significant result was reported). If no age group is indicated beside the finding, then all age groups were affected.

† The scoring system used in the risk of bias assessment is described in the methods section.

Abbreviations: Body mass index (BMI), cardiovascular disease (CVD), chronic kidney disease (CKD), coronary heart disease (CHD), metabolic syndrome (MetS), non-alcoholic fatty liver disease (NAFLD), n/r (not reported), n/s (not specified), peripheral arterial diseases (PAD), type 2 diabetes (T2D), visceral adipose dysfunction (VAD)
