# Supplementary material for: Severe malnutrition or famine exposure in childhood and cardiometabolic non-communicable disease later in life: a systematic review
Source: BMJ Glob Health. 2021 Mar 10;6(3):e003161. doi: 10.1136/bmjgh-2020-003161 (PMC7949429; doi:10.1136/bmjgh-2020-003161)
Supplement: Supplementary data [file bmjgh-2020-003161supp004.pdf]

Online supplementary file 3

Severe malnutrition or famine exposure in childhood on cardiometabolic non-communicable disease risk later in life: a systematic review (Grey K et al., 2020)

Results for outcomes with ≤3 studies examining outcomes

Non-alcoholic fatty liver disease (NAFLD) outcomes

Two Chinese studies examined NAFLD outcomes in famine survivors and both found an increased risk among those exposed in childhood between 1–10y (OR 1.82; 95%CI: 1.35–2.46) and 0–2y (OR 1.26; 95%CI: 1.03–1.55), respectively.[42,55] Wang et al. (2016) found this effect in women only, while all participants in Zheng at al.'s (2017) study were female.[42,55]

Both studies also found similarly increased risk for NAFLD in prenatally exposed women (OR 1.77; 95%CI: 1.22–2.57[42]; OR 1.33; 95%CI: 1.04–1.70[55]).

Physical capacity outcomes

Famine studies

Woo et al. (2010) found no difference in hand-grip strength, average stride length, or walking speed between those who reported a period of famine in childhood compared with those who reported no exposure.[30]

Documented severe malnutrition studies

Two studies examined physical capacity in older children who survived an episode of severe malnutrition. Compared with a well-nourished control group, Bénéfice et al. (1999) found that children who had been both chronically and severely malnourished generally had poorer motor fitness scores with the exception of endurance run scores; however, reduced hand-grip strength in severe malnutrition survivors was the only significant difference between the malnourished groups (p<0.0001).[63] Lelijveld et al. (2016) also found reduced hand-grip strength among severe malnutrition survivors compared with sibling (p=0.005) and community controls (p<0.0001) as well as fewer minutes completed of an exercise test.[9]

Epigenetic & genetic outcomes

Famine studies

Finer et al. (2016) found DNA methylation differences at six of 16 metastable epialleles previously found to be sensitive to maternal nutrition.[21,88] While there were significant methylation differences between post-natal, fetal, and unexposed groups, the predominant differences were among the gestationally-exposed compared to those exposed postnatally or unexposed.[21]

In terms of genetic differences, Rotar et al. (2015) found a shortening of telomere length in Leningrad siege survivors (p<0.0001), with a clear association with the timing of famine in early life. The newborn/infant-exposed group (0–1y) had longer telomeres compared with those exposed between 1–10y and in utero.[38]

Documented severe malnutrition studies

Sheppard et al. (2017) found differences between marasmus and kwashiorkor survivors in DNA methylation near 63 genes in skeletal muscle related to cardiovascular pathways, glucose metabolism, musculoskeletal growth, and body size and composition. However, this study did not include a control group to allow for comparisons with those not exposed to severe malnutrition.[68]

Chronic kidney disease outcomes

Wang et al. (2018) found no association between famine exposure in childhood (1–10y) (OR 1.23; 95%CI: 0.52–2.90) or adolescence/young adulthood (OR 1.18; 95%CI: 0.39–3.59) and chronic kidney disease compared with unexposed controls among a female sample.[45]

Thyroid function outcomes

Zheng et al. (2019) found that compared with non-exposed controls, postnatally exposed participants had lower free thyroxine and higher thyroid stimulation hormone ($p<0.05$). There was no difference in thyroid autoimmune antibodies, heart rate or BMR between groups. Famine exposure did not affect either the number or maximal diameters of thyroid nodules or the TI-RADS score of thyroid nodules.[56]

Metabolomics outcomes

Bourdon et al. (2019) found no differences in metabolite profiles (194 metabolites) between severe malnutrition survivors and sibling and community controls. However, current stunting was associated with IGF-1 ($p<0.0001$) and the relationship was modulated by having experienced severe malnutrition (B=17.4, partial R^2=2.8%, p=0.025). Metabolites were not associated with anthropometric recovery after severe malnutrition nor with severity or type of severe malnutrition.[65]
